# Lymphatic-Immune Microenvironment Engineering via Rapamycin-Eluting Nanofibers for Meningeal Repair and Neuroprotection in Traumatic Brain Injury Rats

**DOI:** 10.34133/research.1415

**Published:** 2026-09-14

**Authors:** Liyuan Zhong, Yu You Huang, Jiaxuan Xie, Xiaofei Han, Yumin Luo, Hanqi Liu, Chunhao Yu, Yi Ming Huang, Yufeng Zheng, Hao Sun, Qing Liu, Zhi Liu, Di Wu, Xunming Ji, Miaowen Jiang, Ming Li

**Affiliations:** ^1^ Department of Neurology and Institute of Cerebrovascular Diseases Research, Xuanwu Hospital of Capital Medical University, Beijing 100053, China.; ^2^ Beijing Institute of Brain Disorders, Capital Medical University, Beijing 100069, China.; ^3^ Neuro-Cardiovascular Research Center, Xuanwu Hospital, Capital Medical University, Beijing 100053, China.; ^4^ AMT Medical Inc., Beijing 102629, China.; ^5^ School of Materials Science and Engineering, Peking University, Beijing 100083, China.; ^6^ Emergency Department, Xuanwu Hospital of Capital Medical University, Beijing 100053, China.

## Abstract

While current dural repair patches achieve mechanical sealing to prevent cerebrospinal fluid leakage, they fail to address the critical pathological cascades of traumatic brain injury, including structural disruption of meningeal lymphatic vessels and dysregulation of neuroimmune responses. Here, we break this therapeutic stalemate through lymphatic-immune microenvironment engineering using rapamycin-eluting poly(l-lactide-*co*-ε-caprolactone) nanofibers. This bioactive patch exerts its therapeutic effects through 3 mechanisms: (a) restoring immune homeostasis by modulating the T helper 17/regulatory T cell balance; (b) morphologically normalizing the meningeal lymphatic network; and (c) preventing cerebral tissue adhesion by suppressing fibroblasts proliferation. In rat traumatic brain injury models, 4% rapamycin-loaded poly(l-lactide-*co*-ε-caprolactone) nanofibers achieved much higher functional recovery and markedly reduced cerebral tissue loss compared to controls. Crucially, it reconstructed the immune microenvironment and normalized the morphology of the meningeal lymphatic system. This study represents a paradigm shift from passive dural closure to active neuroimmunomodulation, offering a neuroprotective therapeutic strategy for traumatic brain injury.

## Introduction

Traumatic brain injury (TBI) remains a major global health burden and imposes a dual neurological crisis: immediate mechanical damage and delayed secondary neurodegeneration driven by immune dysregulation and meningeal lymphatic structural disruption [1–4]. While current clinical sealants (e.g., collagen matrices or silicone sheets) mechanically restore dural continuity, their biological inertness falls short in addressing 3 key pathological processes following TBI: (a) Immune dysregulation drives progressive neuronal apoptosis, (b) fibrotic scarring results from dysregulated fibroblasts activation, and (c) impairment of meningeal lymphatic structure leads to the accumulation of cytotoxic metabolites. This therapeutic trilemma demands a paradigm-shifting material capable of orchestrating structural repair while actively modulating the posttraumatic immune microenvironment and meningeal remodeling.

Emerging evidence highlights immune dysregulation as a central driver of secondary injury progression [5]. T helper 17 cells (Th17 cells) and regulatory T cells (Treg cells), 2 critical subsets of CD4^+^ T cells, play mutually antagonistic roles in immunoregulation [6]. Accumulating evidence suggests that the Th17/Treg cell imbalance may lead to sustained neuroinflammatory activation, further aggravating neuronal damage and functional impairment [1,2,6]. In addition, Th17 cell infiltration has been found to promote fibrosis [7]. Simultaneously, impaired meningeal lymphatic vessels contribute to the accumulation of cytotoxic metabolites, further aggravating neural damage [3,4]. Rapamycin (RAPA), a macrolide compound and potent mammalian target of rapamycin (mTOR) inhibitor, exhibits well-characterized immunomodulatory, anti-inflammatory, and antifibrotic effects by modulating Th17/Treg cell balance and suppressing fibroblasts proliferation [8–10]. However, systemic administration of RAPA is constrained by off-target toxicity and low bioavailability, limiting its therapeutic application in TBI.

**Fig. 1.**
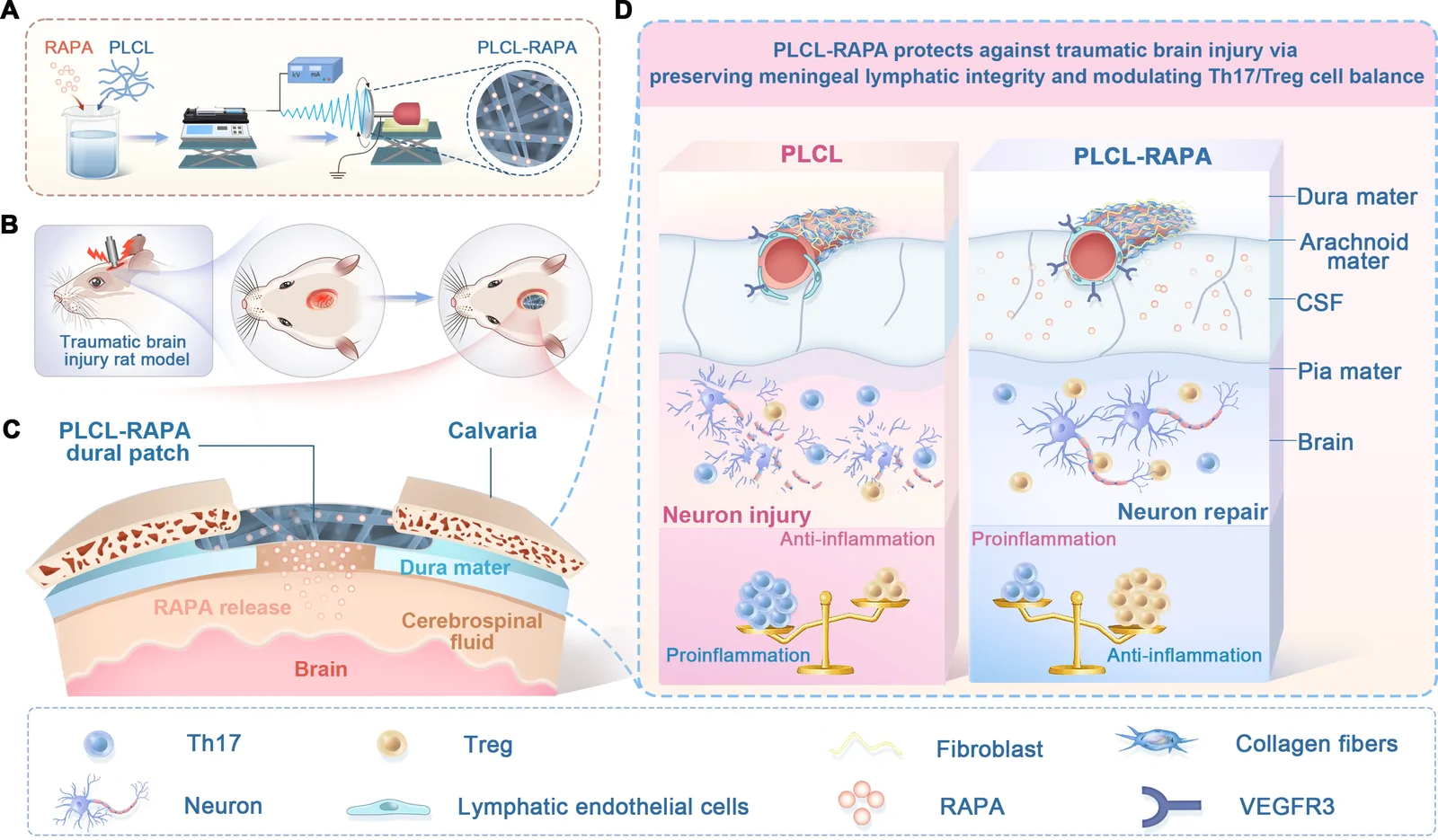
Illustration of poly(l-lactide-*co*-ε-caprolactone) (PLCL) loaded with various concentrations of rapamycin (RAPA) nanofibrous membrane preparation via electrospinning, the animal study procedure, and the proposed brain repair mechanism. Schematic diagram of (A) RAPA-loaded PLCL nanofibrous membranes preparation process, (B) traumatic brain injury rat model establishment, and (C) PLCL-RAPA patch application and RAPA release for dural repair following traumatic brain injury. (D) Schematic hypothesis of the neuroprotective mechanisms of PLCL-RAPA in traumatic brain injury (TBI) through the maintenance of meningeal lymphatic vessel integrity and function, as well as the modulation of the T helper 17/regulatory T cell (Th17/Treg cell) balance (PLCL, control group; PLCL-RAPA, experimental group).

To address these limitations, as detailed in Fig. 1, we developed a bioactive electrospun nanofibrous membrane composed of poly(l-lactide-*co*-ε-caprolactone) (PLCL) loaded with various concentrations of RAPA (PLCL-RAPA). We hypothesized that this multifunctional membrane could restore immune homeostasis, prevent brain tissue adhesion, maintain the integrity of meningeal lymphatic vessels, and ultimately enhance neurological recovery following TBI. To validate this hypothesis, we conducted a comprehensive series of in vitro experiments. These experiments included immunofluorescence staining of T lymphocytes, proliferation assay of NIH/3T3 fibroblasts, tube formation analysis of human lymphatic endothelial cells (HLECs), and terminal deoxynucleotidyl-transferase-mediated deoxyuridine triphosphate nick end labeling (TUNEL) assay in SH-SY5Y cells subjected to oxygen-glucose deprivation/reperfusion (OGD/R). In vivo validation in a rat TBI model included behavioral tests (foot fault and Y-maze), targeted metabolomics (assessing brain energy metabolism and tryptophan pathway in cerebrospinal fluid [CSF]), multiparametric immunofluorescence, and flow cytometry. These analyses evaluated dura mater regeneration, meningeal lymphatic vessel preservation, neuronal repair, and immune microenvironment regulation.

Through a series of comprehensive in vivo and in vitro experiments, we demonstrated that our engineered multifunctional membrane promotes post-TBI neurological recovery. The innovative contributions of this study manifest across 3 synergistic dimensions: functional duality (meningeal repair/neuroprotection integration), mechanistic revelation (mTOR-mediated meningeal–lymphatic–immune triad), and methodological improvement (immune-centric neuroprotection evaluation framework). This work transcends traditional “seal-and-separate” approaches by introducing a bioactive interface that actively negotiates neuroimmune interactions, opening new avenues for the management of TBI and the design of neuroprotective materials.

## Results

### Characterization of PLCL-RAPA

**Fig. 2.**
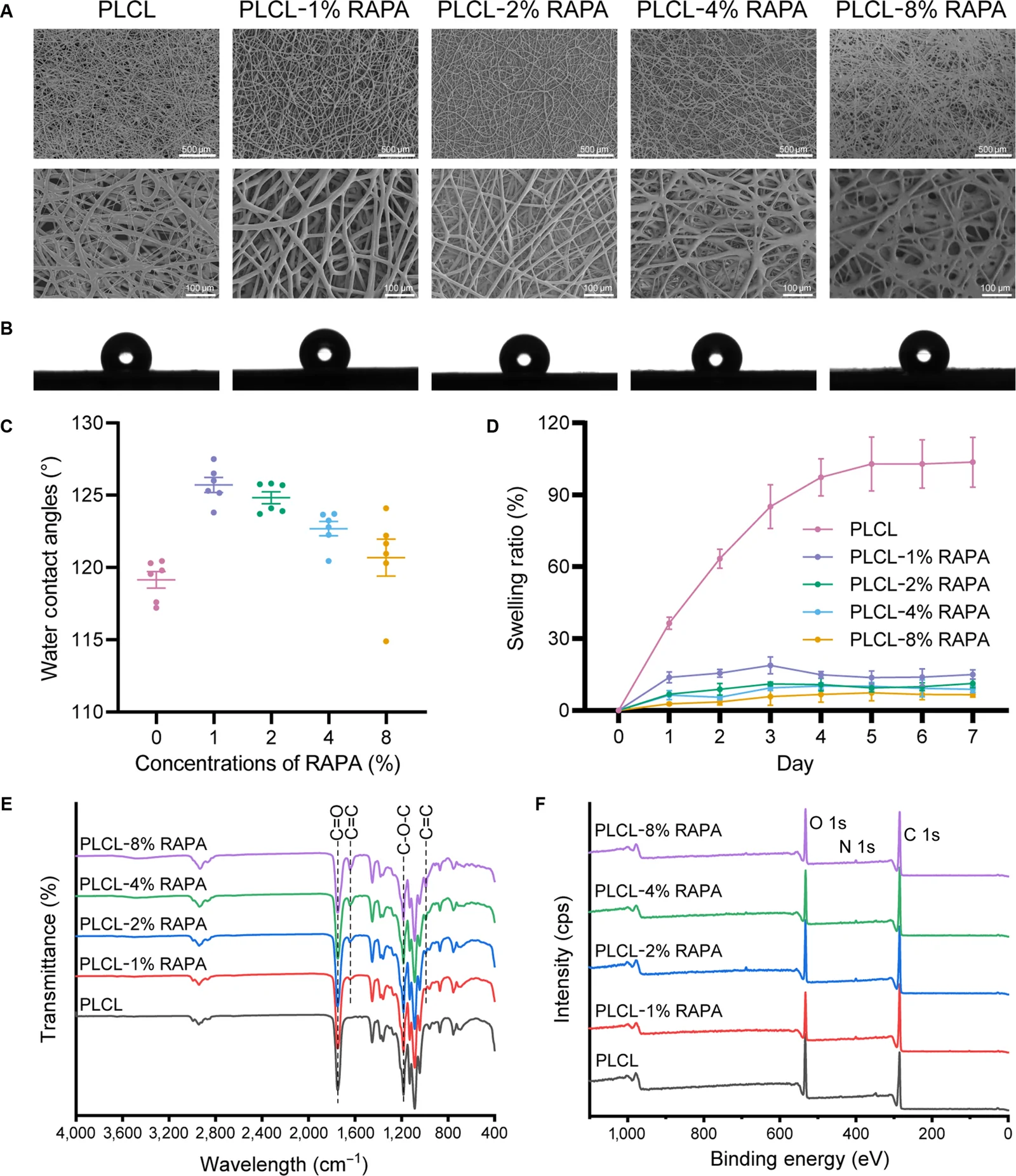
Physicochemical characterization of electrospun PLCL-RAPA nanofibrous membranes with varying RAPA content (0 to 8 wt%). (A) Scanning electron microscopy (SEM) images. (B) Representative water contact angle images. (C) Quantitative analysis, (D) Swelling kinetics profiles. (E) Fourier transform infrared (FTIR) spectra, and (F) X-ray photoelectron spectroscopy (XPS) survey spectra. Data are presented as the means ± SD and analyzed using ANOVA with Tukey post hoc test.

Structural and surface characterization of the electrospun PLCL membranes with varying RAPA loading (0 to 8 wt%) confirmed successful fabrication of uniform nanofibrous scaffolds. Scanning electron microscopy (SEM) analysis demonstrated that RAPA incorporation did not significantly alter the fiber morphology, with all groups maintaining consistent fiber diameters and interconnected porous architectures (Fig. 2A). This structural preservation is crucial for ensuring mechanical stability while facilitating cell infiltration and nutrient transport. Surface wettability analysis revealed concentration-dependent modulation of hydrophilicity (Fig. 2B and C). Interestingly, while pure PLCL membranes exhibited a water contact angle of 119.15° ± 1.40°, RAPA incorporation induced a more hydrophobic surface but with progressive decrease in contact angles to 121.84° ± 1.45° at 8% loading. This apparent paradox, where a hydrophobic drug (RAPA) decreases membrane wettability, can be explained by 2 competing mechanisms: (a) increased surface roughness from drug-polymer phase separation and (b) hydrogen bonding between water molecules and polar groups in RAPA’s molecular structure (particularly the nitrogen and oxygen atoms in its macrolide ring).

Swelling behavior analysis demonstrated RAPA’s influence on hydration kinetics (Fig. 2D). Drug-loaded membranes reached swelling equilibrium within 72 h, whereas pure PLCL controls required to 120 h. The equilibrium swelling ratio decreased from 103.50% ± 18.04% (PLCL) to 6.59% ± 1.67% (PLCL-8% RAPA), likely due to RAPA’s hydrophobic nature restricting water penetration and its potential plasticizing effect on the polymer matrix. Molecular characterization via Fourier transform infrared (FTIR) confirmed successful RAPA incorporation while maintaining PLCL’s structural integrity (Fig. 2E). The persistent carbonyl stretching vibration at 1,753 cm^−1^ indicated undisturbed ester linkages in PLCL, whereas new peaks at 1,645 cm^−1^ (C=C stretching) and 991 cm^−1^ (C–H bending) were assigned to characteristic RAPA functional groups [11]. X-ray photoelectron spectroscopy (XPS) analysis provided further evidence of RAPA incorporation through the appearance of a nitrogen 1s peak (399.8 eV) in drug-loaded membranes (Fig. 2F), which was absent in pure PLCL spectra.

The release kinetics demonstrated a positive correlation between RAPA release rate and its loading content in PLCL, reaching a plateau phase by day 10 with cumulative release exceeding 90% (Fig. S1A). Hemolysis assays revealed that PLCL-RAPA with 0 to 4 wt% RAPA induced ~1% hemolysis, whereas the 8 wt% formulation reached ~13% (Fig. S1B).

### PLCL-RAPA modulated multiple cellular behaviors

#### Cytotoxicity of PLCL-RAPA

**Fig. 3.**
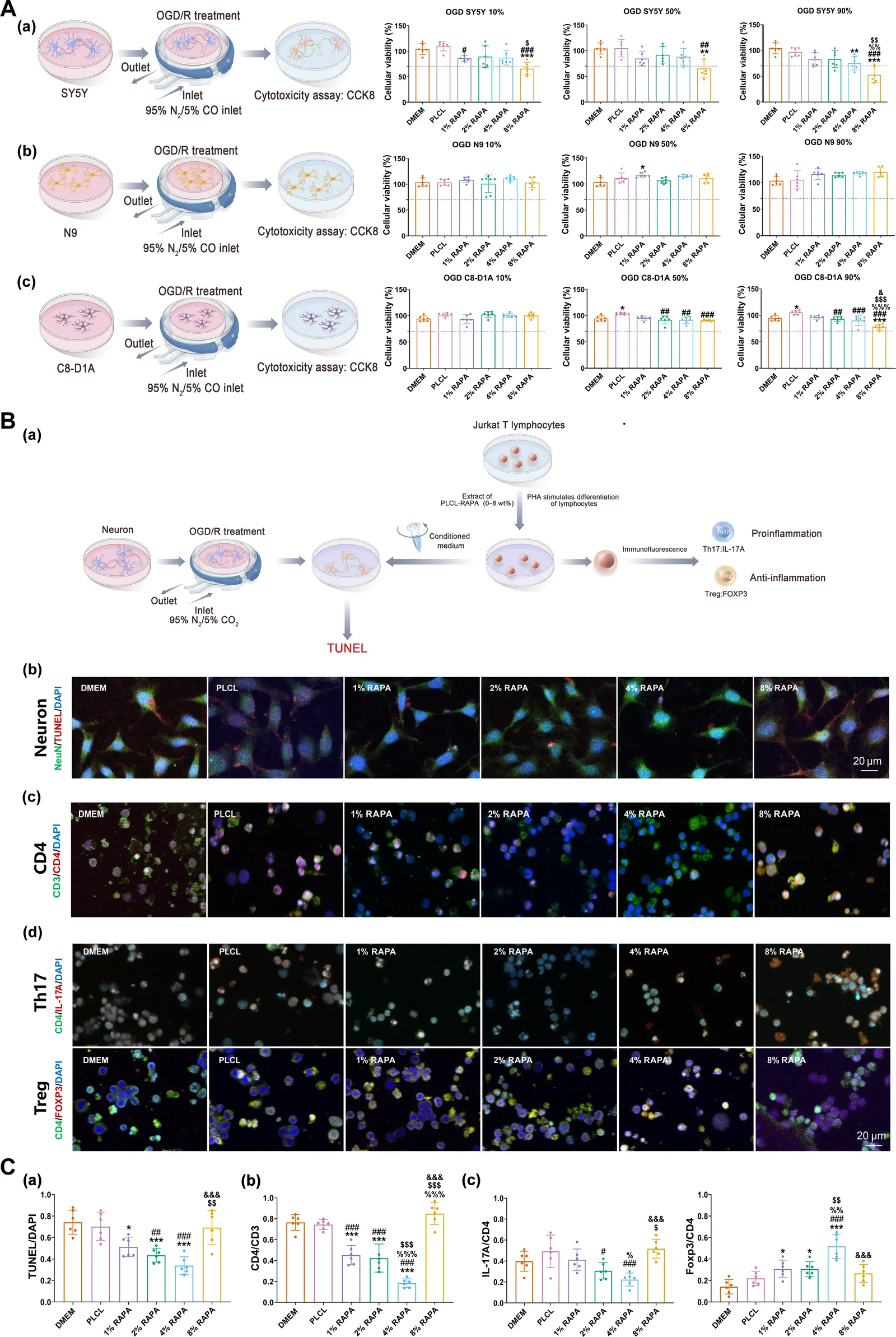
Cytotoxicity assessment and neuroprotective mechanisms of PLCL-RAPA nanofibers against oxygen-glucose deprivation/reperfusion (OGD/R) injury. (A) Cytotoxicity assay of electrospun PLCL-RAPA nanofibrous membranes with varying RAPA content (0 to 8 wt%) on (a) neuronal (SY5Y), (b) microglial (N9), and (c) astrocytic (C8-D1A) cell lines subjected to OGD/R. (B) Investigation of the neuroprotective effects of PLCL-RAPA nanofibers against OGD/R injury and the underlying mechanisms: (a) schematic diagram of the experimental procedure for SH-SY5Y cells and Jurkat T lymphocytes, (b) representative terminal deoxynucleotidyl-transferase-mediated deoxyuridine triphosphate nick end labeling (TUNEL) staining images of SH-SY5Y cells, and (c and d) representative immunofluorescence staining images of Jurkat T lymphocytes. (C) Statistical analysis of (a) the percentage of TUNEL-positive cells, (b) the proportion of CD4^+^ (CD3^+^CD4^+^) T cells, and (c) the proportion of T helper 17 cells (Th17 cells) (CD4^+^IL-17A^+^) and regulatory T cells (Treg cells) (CD4^+^Foxp3^+^). Data are presented as the means ± SD and analyzed using ANOVA with Tukey post hoc test (*P < 0.05, **P < 0.01, and ***P < 0.001 versus Dulbecco’s modified Eagle’s medium [DMEM] group; #*P* < 0.05, ##*P* < 0.01, and ###*P* < 0.001 versus PLCL group; %*P* < 0.05, %%*P* < 0.01, and %%%*P* < 0.001 versus 1% RAPA group; $*P* < 0.05, $$*P* < 0.01, and $$$*P* < 0.001 versus 2% RAPA group; &*P* < 0.05 and &&&*P* <0.001 versus 4% RAPA group). DAPI, 4′,6-diamidino-2-phenylindole.

Comprehensive cytotoxicity evaluation revealed distinct cellular responses to PLCL-RAPA extracts (Fig. 3A and Fig. S2). SH-SY5Y neuronal cells demonstrated concentration-dependent sensitivity, with PLCL, 1% RAPA, and 2% RAPA extracts maintaining >70% viability across all concentrations (10% to 90%) under both normoxic and OGD/R conditions (ISO 10993-5 compliant). Notably, the 4% RAPA formulation showed selective cytotoxicity (viability < 70%) at ≥50% extract concentration under normoxic conditions, while the 8% RAPA extract exhibited significant toxicity at all concentrations in normal and OGD/R-treated neurons [Fig. 3A(a) and Fig. S2A].

Microglial cells (N9) displayed remarkable resilience, maintaining >70% viability regardless of RAPA loading (0% to 8%) or extract concentration, suggesting inherent resistance to RAPA’s immunomodulatory effects [Fig. 3A(b) and Fig. S2B].

C8-D1A astrocytes maintained >70% viability regardless of RAPA loading (0% to 8%) or extract concentration under OGD/R conditions [Fig. 3A(c)]. However, C8-D1A astrocytes showed complex concentration thresholds under normoxic conditions: All formulations were biocompatible at 10% extract concentration (Fig. S2C); the 8% RAPA formulation showed selective cytotoxicity (viability < 70%) at ≥50% extract concentration (Fig. S2C); the 4% RAPA extract exhibited significant toxicity at 90% extract concentration (Fig. S2C).

These findings collectively demonstrate that while lower RAPA concentrations (1% to 2%) ensure broad biocompatibility, the 4% loading offers a potentially optimal balance between safety and therapeutic effect, particularly for neuronal and astrocyte populations.

### PLCL-RAPA provides neuroprotection via modulating Th17/Treg cell balance

To investigate the neuroprotective and immunomodulatory potential of RAPA-loaded membranes, we established an in vitro ischemia model (Fig. 3B). Jurkat T lymphocytes were initially treated with PLCL-RAPA extract (0 to 8 wt%) for 24 h to generate conditioned media, which were subsequently added to OGD/R SH-SY5Y neuronal cultures [Fig. 3B(a)]. The neuroprotective capacity was assessed through immunofluorescence and TUNEL staining [Fig. 3B(b) and C(a)]. Conditioned media from 1% to 4% RAPA-treated groups exhibited dose-dependent neuroprotective effects, reducing neuronal apoptosis (TUNEL^+^ cells) compared to the Dulbecco’s modified Eagle’s medium (DMEM) control group [*P* < 0.05 and *P* < 0.001; Fig. 3B(b) and C(a)]. The 8% RAPA media failed to provide significant protection, confirming the therapeutic concentration threshold [Fig. 3B(b) and C(a)].

Multiparametric immunofluorescence analysis revealed that conditioned media from 1% to 4% RAPA membranes induced significant immunophenotypic shifts in Jurkat cells compared to PLCL controls [Fig. 3B(c and d) and C(b and c)]: (a) reduction in CD3^+^/CD4^+^ populations [*P* < 0.001; Fig. 3B(c) and C(b)]; (b) down-regulation of Th17 cell subsets [*P* > 0.05, *P* < 0.05, and *P* < 0.001; Fig. 3B(d) and C(c)]; and (c) expansion of Treg cells [*P* > 0.05 and *P* < 0.001; Fig. 3B(d) and C(c)]. These immunomodulatory effects were concentration dependent and absent in the 8% RAPA group [Fig. 3B(c and d) and C(b and c)].

### Effect of PLCL-RAPA on fibroblasts and lymphatic endothelial cells

**Fig. 4.**
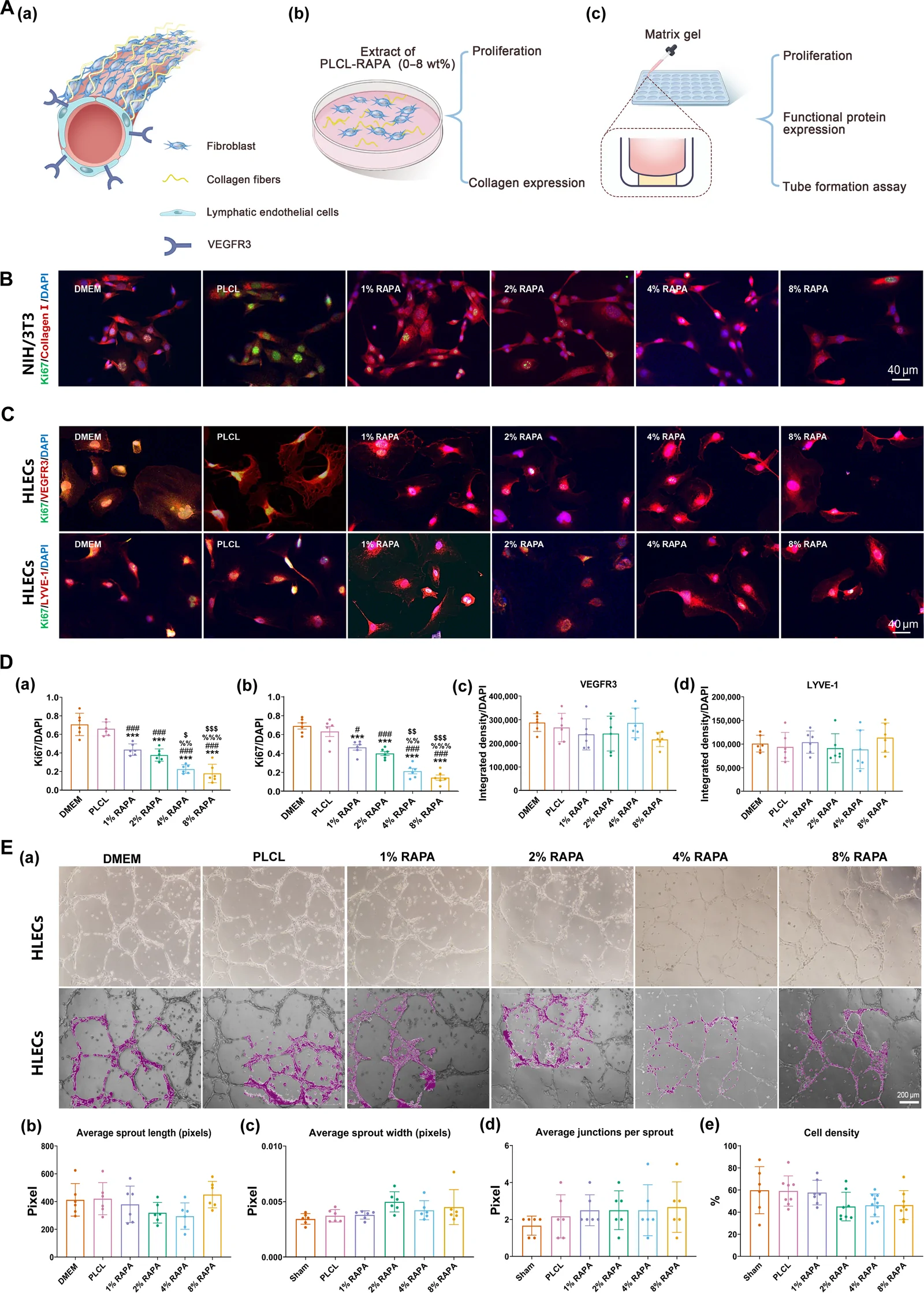
PLCL-RAPA inhibits proliferation of fibroblasts and lymphatic endothelial cells in vitro. (A) Schematic diagram of the experimental procedure: (a) schematic diagram of lymphatic vessel and fibroblasts, (b) experimental design of fibroblasts, (c) experimental design of human lymphatic endothelial cells. (B to E) Effect of electrospun PLCL-RAPA nanofibrous membranes with varying RAPA content (0 to 8 wt%) on fibroblasts and human lymphatic endothelial cells. (B and C) Representative Ki67 immunofluorescence of (B) fibroblasts and (C) lymphatic endothelial cells. (D) Statistical analysis of the percentage of Ki67-positive (a) fibroblasts and (b) human lymphatic endothelial cells; statistical analysis of (c) VEGFR3-integrated density and (d) LYVE-1-integrated density. (E) (a) Representative images of tube formation, (b to e) statistical analysis of tube formation, (b) average sprout length of each group, (c) average sprout width of each group, (d) average junctions per sprout of each group, and (e) cell density of each group. Data are presented as the means ± SD and analyzed using ANOVA with Tukey post hoc test (***P < 0.001 versus Dulbecco’s modified Eagle’s medium [DMEM] group; #*P* < 0.05 and ###*P* < 0.001 versus PLCL group; %%*P* < 0.01 and %%%*P* < 0.001 versus 1% RAPA group; $*P* < 0.05, $$*P* < 0.01, and $$$*P* < 0.001 versus 2% RAPA group).

Our findings demonstrate RAPA’s multifaceted dose-dependent effects on dural repair processes (Fig. 4). Quantification of Ki67^+^/collagen I^+^ double-positive cells revealed a progressive reduction in fibroblasts proliferation with increasing RAPA concentration (1% to 8% wt) [Fig. 4B and D(a)], suggesting potent antifibrotic activity that may prevent postsurgical adhesions. Similarly, lymphatic endothelial cell analysis showed (a) decrease in Ki67^+^/vascular endothelial growth factor receptor 3-positive (VEGFR3^+^) proliferating cells, while maintaining stable VEGFR3^+^ populations [Fig. 4C and D(b and c)]; (b) parallel reduction in Ki67^+^/lymphatic vessel endothelial hyaluronan receptor 1-positive (LYVE-1^+^) cells without affecting LYVE-1 expression [Fig. 4C and D(b and d)]. Notably, tube formation assays confirmed that lymphatic endothelial cells exhibited no significant differences in branching points or total tube length (Fig. 4E). These results establish that RAPA selectively inhibits fibroblasts and lymphatic endothelial proliferation while maintaining critical lymphatic markers and angiogenic capacity—an ideal combination for preventing fibrosis while preserving normal meningeal lymphatic structure during dural repair. In addition, lymphocyte conditioned medium from the PLCL-4% RAPA group increased the proportion of forkhead box P3-positive (FOXP3^+^)/CD4^+^ lymphocytes and reduced interleukin-17A-positive (IL-17A^+^)/CD4^+^ lymphocytes, indicating a shift toward a less inflammatory lymphocyte phenotype. When applied to SVEC4-10 cells, this conditioned medium enhanced lymphatic endothelial tube formation and increased LYVE-1 expression, whereas recombinant IL-17A partially attenuated these effects without significantly altering Ki67 expression (Fig. S3).

### Implantation of PLCL-RAPA in TBI rats

#### PLCL-RAPA provides neuroprotection, inhibits proliferation of fibroblasts, and preserves meningeal lymphatic integrity in TBI rats

**Fig. 5.**
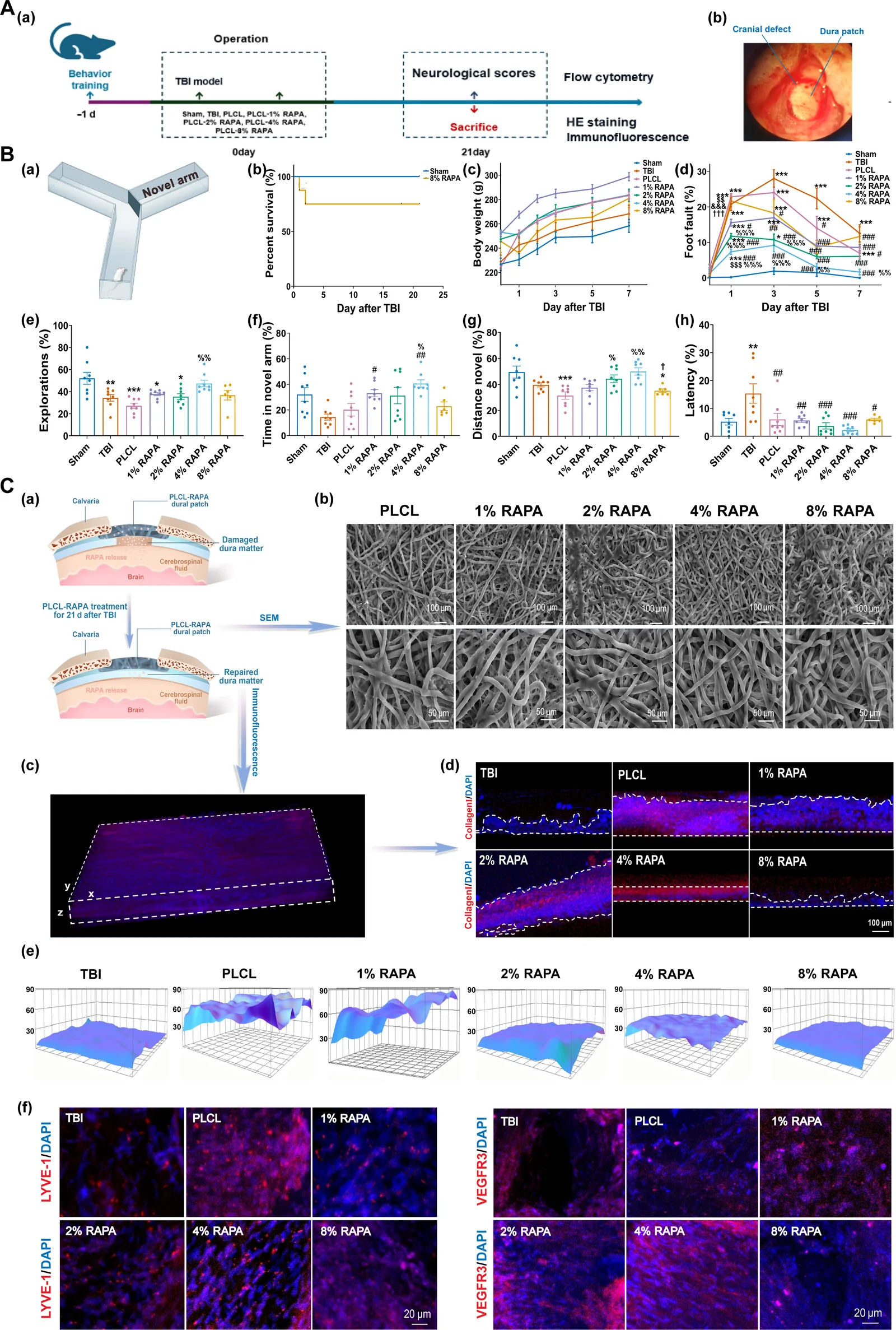
PLCL-RAPA provides neuroprotection, inhibits proliferation of fibroblasts, and preserves meningeal lymphatic integrity in traumatic brain injury rats. (A) Schematic diagram: (a) schematic illustration of experimental timeline, and (b) surgical photogram showing the application of a PLCL-RAPA patch for dural repair following traumatic brain injury. (B) PLCL-RAPA provides neuroprotection in traumatic brain injury rats: (a) schematic diagram of Y-maze, (b) survival curve, (c) weight changes, (d) foot fault test, and (e to h) Y-maze test. (C) PLCL-RAPA inhibits proliferation of fibroblasts and preserves the structural integrity of lymphatic vessels in traumatic brain injury rats: (a) schematic diagram of the repair process for damaged dura mater, (b) scanning electron microscopy (SEM) images of PLCL-RAPA after 21 d in vivo, (c) schematic of z-stack confocal fluorescence, (d) *z*-axis thickness projection of fibroblasts stained with collagen I, and (e and f) representative immunofluorescence images of lymphatic vessel. Data are presented as the means ± SD and analyzed using ANOVA with Tukey post hoc test (*P < 0.05, **P < 0.01, and ***P < 0.001 versus sham group; #*P* < 0.05, ##*P* < 0.01, and ###*P* < 0.001 versus traumatic brain injury [TBI] group; %*P* < 0.05%, %%*P* < 0.01, and %%%*P* < 0.001 versus PLCL group; $$*P* < 0.01 and $$$*P* < 0.001 versus 1% RAPA group; &&&*P* < 0.001 versus 2% RAPA group; †*P* < 0.05 and †††*P* < 0.001 versus 4% RAPA group).

Following uniform decompressive craniectomy (control animals received craniectomy alone; treatment groups received craniectomy plus implantation), our comprehensive in vivo assessment revealed concentration-dependent therapeutic effects of RAPA-loaded membranes in TBI treatment (Fig. 5 and Fig. S4). The survival analysis demonstrated a critical toxicity threshold, with the 8% RAPA group exhibiting significantly higher mortality (25%) compared to other groups [*P* < 0.05; Fig. 5B(b)], establishing an upper safety limit for RAPA concentration. Behavioral tests showed marked improvements in neurological function: The PLCL-4% RAPA group displayed a reduction in foot fault ratio versus TBI controls [*P* < 0.001; Fig. 5B(d)], indicating restored motor coordination. Cognitive assessment in the Y-maze revealed improved performance in the PLCL-4% RAPA group compared with TBI controls, characterized by increased novel arm exploration [*P* > 0.05; Fig. 5B(e)], longer exploration duration [*P* < 0.01; Fig. 5B(f)], greater movement distance [*P* > 0.05; Fig. 5B(g)], and reduced exploration latency [*P* < 0.05; Fig. 5B(h)].

Electron microscopy analysis demonstrated that the swelling characteristics of the RAPA-loaded membranes remained stable without notable changes after 21 d of implantation in rats with TBI [Fig. 5C(b)].

Immunofluorescence analysis of dural tissues revealed significant therapeutic effects of our RAPA-loaded membranes [Fig. 5C(d to g)]. Compared to TBI controls, both the PLCL and 4% RAPA groups demonstrated enhanced dural regeneration, as evidenced by an increase in collagen I^+^ cells [Fig. 5C(d) and Fig. S5], indicating active extracellular matrix deposition. However, the 4% RAPA group showed superior tissue remodeling characteristics, exhibiting (a) a reduction in dural thickness compared to PLCL controls, suggesting prevention of pathological fibrosis [Fig. 5C(d and e)], and (b) significant restoration of the lymphatic network, accompanied by respective increases in LYVE-1^+^ and VEGFR3^+^ cells [Fig. 5C(f and g)]. Notably, the lymphatic endothelial cells in the 4% RAPA group displayed more organized spatial distribution and elongated morphology compared to the disorganized patterns observed in PLCL controls [Fig. 5C(f and g)], demonstrating RAPA’s dual capacity to simultaneously promote dural regeneration while maintaining physiological lymphatic architecture. These findings suggest that 4% RAPA loading optimally balances extracellular matrix deposition with antifibrotic activity and lymphatic network preservation during dural repair.

#### PLCL-RAPA suppresses the kynurenine pathway and increases serotonin

**Fig. 6.**
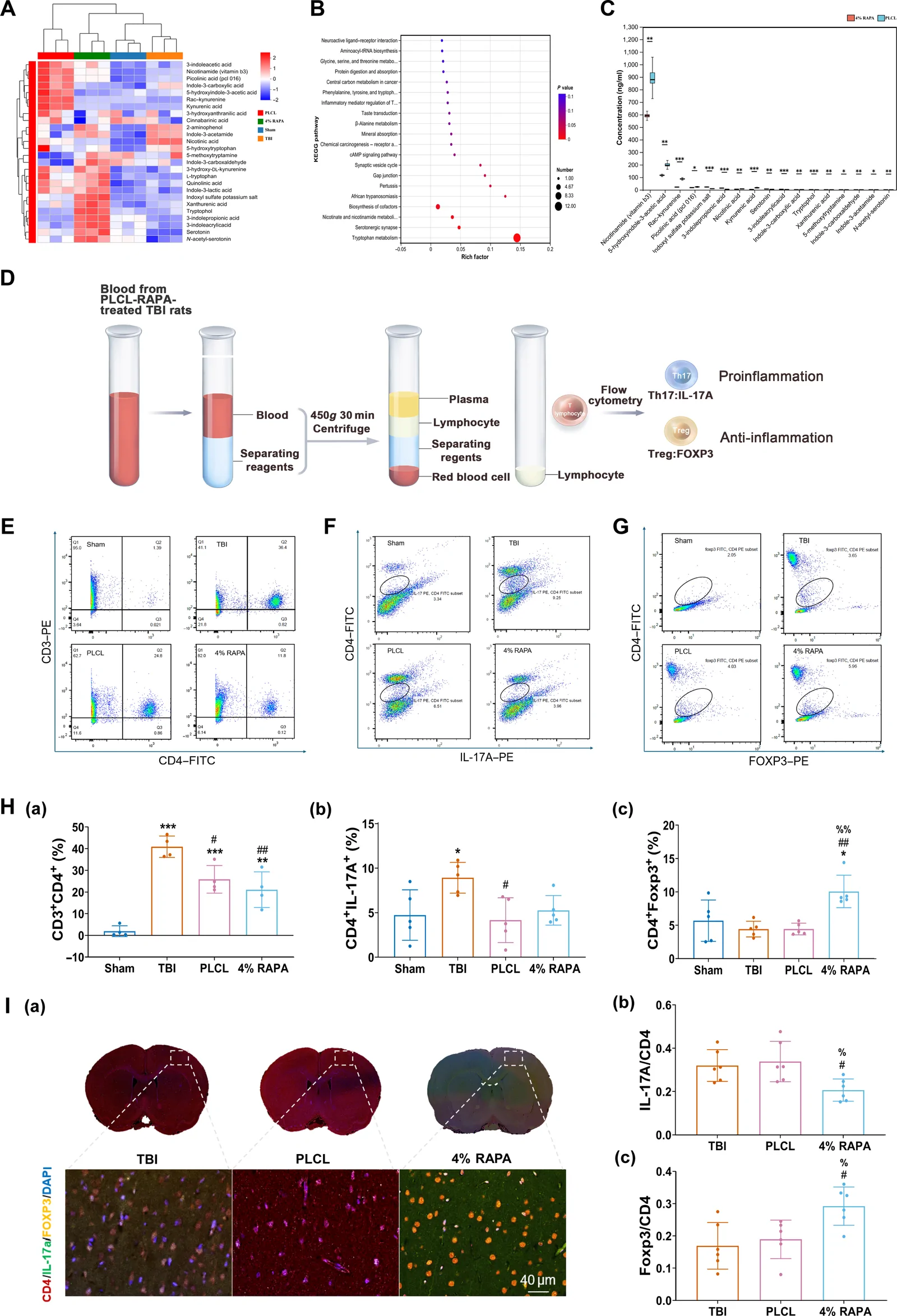
PLCL-RAPA suppresses kynurenine pathway, increases serotonin, and modulates T helper 17/regulatory T cell (Th17/Treg cell) balance in traumatic brain injury rats. (A) Heatmap analysis of differential metabolites in cerebrospinal fluid (CSF). (B) The enrichment dot bubble chart of the Kyoto Encyclopedia of Genes and Genomes (KEGG) pathways enrichment analysis of CSF (PLCL versus 4% RAPA). (C) Bar chart of the KEGG pathways enrichment analysis of CSF (PLCL versus 4% RAPA). (D) Schematic diagram of lymphocytes isolation. (E) The flow cytometry scatter plot of CD4^+^ T cells in lymphocytes. (F) The flow cytometry scatter plot of Th17 cells in CD4^+^ T lymphocytes. (G) The flow cytometry scatter plot of Treg cells in CD4^+^ T lymphocytes. (H) Statistical analysis of the percentages of (a) the CD4^+^ populations, (b) the Th17 cell populations, and (c) the Treg cell populations. (I) (a) Representative immunofluorescence images Th17 cells and Treg cells and (b and c) statistical analysis of the percentage of (b) Th17 cells and (c) Treg cells. Data are presented as the means ± SD and analyzed using ANOVA with Tukey post hoc test (*P < 0.05, **P < 0.01, and ***P < 0.001 versus sham group; #*P* < 0.05 and ##*P* < 0.01 versus traumatic brain injury [TBI] group; %*P* < 0.05% and %%*P* < 0.01 versus PLCL group). cAMP, cyclic adenosine monophosphate; PE, phycoerythrin; FITC, fluorescein isothiocyanate.

Targeted metabolomic analysis revealed marked remodeling of the tryptophan metabolism network in CSF following RAPA treatment. Key findings included the following: down-regulation of kynurenine (log_2_ fold change [log_2_FC] = –2.01, false discovery rate < 0.001), along with nicotinic acid (log_2_FC = –0.69) and nicotinamide (log_2_FC = –0.60), suggesting inhibition of the kynurenine–nicotinamide adenine dinucleotide (oxidized form) (NAD^+^) axis (Fig. 6A to C); and up-regulation of serotonin (log_2_FC = +0.96, false discovery rate = 0.016), indicating a metabolic shift toward the neuroprotective serotonergic branch (Fig. 6A to C). Picolinic acid, an immunomodulatory by-product of the kynurenine pathway, was also down-regulated (log_2_FC = –0.42), suggesting reduced microglial activation (Fig. 6A and C). These results reflect an inflammatory metabolic phenotype, characterized by a shift in tryptophan catabolism away from the excitotoxic kynurenine axis and toward the serotonin and NAD^+^ pathways. To further verify these results, we determined the protein expression of kynurenine 3-monooxygenase (KMO), a key regulatory enzyme in the kynurenine pathway, in rat brain tissues by Western blotting. Compared with the sham group, KMO levels were increased in both the TBI and PLCL groups. In contrast, the 4% RAPA-treated group exhibited decreased KMO expression relative to the TBI and PLCL groups (Fig. S6A and B). This suggests that RAPA reprograms tryptophan metabolism.

#### PLCL-RAPA modulates Th17/Treg cell balance

Western blot analysis showed that phosphorylated mTOR (p-mTOR) levels were elevated in both the TBI and PLCL groups compared to the sham group. In contrast, treatment with 4% RAPA reduced p-mTOR expression relative to the TBI and PLCL groups (Fig. S6C and D).

Flow cytometry analysis revealed profound alterations in T cell subpopulations following TBI and their subsequent modulation by RAPA treatment (Fig. 6). Compared to sham controls, both TBI and PLCL groups exhibited marked immunological disturbances characterized by (a) an elevation in CD3^+^/CD4^+^ T cell ratio, indicating generalized T cell activation [Fig. 6E and H(a)]; (b) a polarized Th17 cell response evidenced by an increase in CD4^+^/IL-17A^+^ cells [Fig. 6F and H(b)]; and (c) compromised immunoregulation demonstrated by a decrease in CD4^+^/FOXP3^+^ Treg cells [Fig. 6G and H(c)]. Remarkably, the 4% RAPA treatment effectively normalized these pathological changes, restoring immune homeostasis through (a) reduction in overall T cell activation [CD3^+^/CD4^+^; Fig. 6E and H(a)]; (b) suppression of Th17 cell responses [Fig. 6F and H(b)]; and (c) expansion of Treg cells [Fig. 6G and H(c)]. Immunofluorescence validation confirmed these findings, showing significantly higher ratios of CD4^+^/FOXP3^+^ (Treg) cells in the 4% RAPA group compared to TBI and PLCL controls [*P* < 0.05; Fig. 6I(a and c)], demonstrating RAPA’s capacity to simultaneously enhance protective immunity and suppress detrimental inflammatory responses in the TBI microenvironment.

#### PLCL-RAPA attenuates brain atrophy, reduces adhesion to brain tissue, and alleviates neuronal loss in TBI rats

**Fig. 7.**
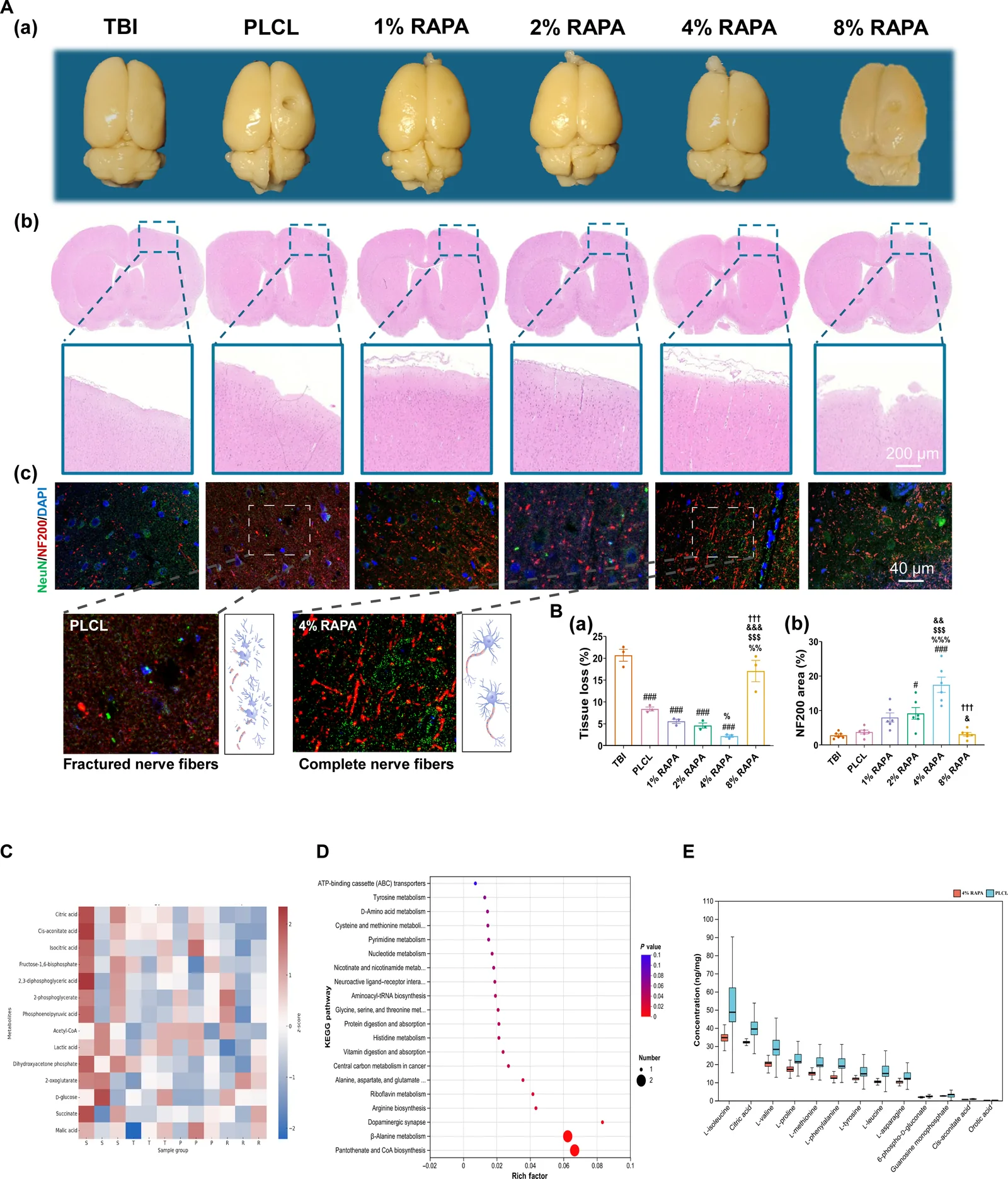
PLCL-RAPA attenuates brain atrophy, reduces adhesion to brain tissue, and alleviates neuronal loss in traumatic brain injury rats. (A) Representative images of (a) brain tissue, (b) hematoxylin and eosin (HE) staining, and (c) immunofluorescence staining. (B) Quantitative analysis of (a) brain tissue loss volume and (b) neurofilament 200 (NF200) immunofluorescence staining. (C) Heatmap of differential metabolites in brain tissue (S, sham group; T, TBI group; P, PLCL group; R, 4% RAPA group). (D) The enrichment dot bubble chart of the Kyoto Encyclopedia of Genes and Genomes (KEGG) pathways enrichment analysis of brain tissue (PLCL versus 4% RAPA). (E) Bar chart of the KEGG pathways enrichment analysis of brain tissue (PLCL versus 4% RAPA). Data are presented as the means ± SD and analyzed using ANOVA with Tukey post hoc test (#*P* < 0.05 and ###*P* < 0.001 versus traumatic brain injury [TBI] group; %*P* < 0.05 and %%%*P* < 0.001 versus PLCL group; $$$*P* < 0.001 versus 1% RAPA group; &*P* < 0.05, &&*P* < 0.01, and &&&*P* < 0.001 versus 2% RAPA group; †††*P* < 0.001 versus 4% RAPA group).

Histopathological examination demonstrated marked neuroprotection in RAPA-treated groups. While TBI controls showed right hemisphere atrophy and PLCL groups exhibited dural adhesions, RAPA-treated animals (1% to 4%) maintained better preserved brain architecture [Fig. 7A(a)]. Quantitative analysis of hematoxylin and eosin (HE)-stained sections revealed a dose-dependent reduction in neuronal loss at the injury site [1 to 4% RAPA; Fig. 7A(b) and B(a)], contrasting with the greater neuronal loss observed in the 8% RAPA group compared with PLCL controls [*P* < 0.01; Fig. 7A(b) and B(a)]. Immunofluorescence analysis corroborated these observations, demonstrating a significant up-regulation of neurofilament expression in the 4% RAPA group relative to PLCL controls [*P* < 0.001; Fig. 7A(c) and B(b)]. In contrast, the 8% RAPA group exhibited a marked reduction in neurofilament expression compared to the 4% RAPA group [*P* < 0.001; Fig. 7A(c) and B(b)].

Taken together, these findings identify 4% RAPA as the optimal therapeutic concentration, providing (a) significant functional recovery, (b) neural tissue preservation, and (c) prevention of dural adhesions, while clearly demonstrating the neurotoxicity of 8% RAPA concentration through multiple evaluation parameters.

To investigate metabolic dysfunction following TBI, we performed targeted metabolomic profiling of brain tissue. The results revealed that compared to sham animals, the TBI group exhibited significantly reduced levels of citric acid, isocitric acid, and succinic acid, indicative of mitochondrial dysfunction and impaired oxidative phosphorylation (Fig. 7C). This metabolic collapse was accompanied by diminished expression of downstream metabolites in glycolytic and anaplerotic pathways, suggesting a system-wide energy deficit. In the tricarboxylic acid (TCA) cycle, succinate levels were elevated in the 4% RAPA group compared to PLCL (log_2_FC = +0.41), suggesting enhanced mitochondrial electron transport flux. Conversely, citric acid and acetyl–coenzyme A (CoA) levels were modestly reduced in the 4% RAPA group (log_2_FC = –0.15 and –0.45, respectively), potentially reflecting decreased anaplerotic flux into lipid biosynthesis—a hallmark of mTOR inhibition (Fig. 7D and E). Other TCA intermediates such as isocitric acid remained relatively stable between groups (log_2_FC = –0.08; Fig. 7D and E). Energy cofactor measurements showed subtle improvements in the 4% RAPA group: NAD^+^ and adenosine triphosphate (ATP) concentrations were slightly higher compared to 4% PLCL controls (log_2_FC = +0.09 and +0.05, respectively; Fig. 7D and E). These trends indicate that RAPA may promote more efficient oxidative phosphorylation and ATP production by curtailing anabolic demands. In contrast, the PLCL group primarily preserved baseline metabolic patterns without substantially modifying TCA cycle or cofactor levels, consistent with its role as a structural but metabolically inert scaffold. To verify these findings, we conducted Western blot analysis to examine the expression of α-ketoglutarate dehydrogenase (OGDH), a critical enzyme in the TCA cycle, in rat brain tissues. Compared with the sham group, OGDH protein levels were reduced in both the TBI and PLCL groups. In contrast, administration of 4% RAPA resulted in elevated OGDH expression relative to the TBI and PLCL groups (Fig. S6E and F).

## Discussion

DuraGen, a widely used clinical dural substitute, lacks inherent suture strength and demonstrates poor tensile resistance, potentially compromising reliable mechanical closure in large or tension-bearing defects and consequently increasing the risk of postoperative CSF leakage. Previous studies have demonstrated that PLCL exhibits favorable tensile properties [12]. In addition, PLCL has been reported to promote tissue regeneration [13]. Our study introduces a paradigm shift in dural repair by developing an electrospun PLCL-RAPA scaffold that addresses critical limitations of conventional materials that primarily focus on passive barrier functions. As shown in Table 1, the existing dural substitutes emphasize antiadhesion and antibacterial properties [14–21]. The major contributions of this study reside in 3 aspects as follows:

1. Functional duality: A dual-functional dural repair system is prepared that synergistically integrates structural meningeal regeneration with active neuroprotection, which is an effective advancement beyond single-purpose clinical grafts.
2. Mechanistic revelation: A novel therapeutic meningeal–lymphatic–immune triad model is proposed, wherein RAPA-eluting PLCL membranes coordinate structural meningeal repair, normalization of lymphatic morphology, and T cell homeostasis.
3. Methodological improvement: A combined in vitro–in vivo validation approach is established that evaluates neuroprotection through immune remodeling rather than conventional neuron-centric methods.

**Table 1.** Comparison with other studies on the design of dural patch

Animal model	Materials composition	Functional features				Reference
		Modulates Th17/Treg cell balance	Prevents adhesion	Provides neuroprotection	Preserves meningeal lymphatic integrity	
Rats with traumatic brain injury	PLCL/RAPA	√	√	√	√	Our study
Rabbits underwent laminectomy	Electrospun membranes/mitomycin-C/meloxicam	×	√	×	×	[15]
Rabbits with spinal cord injury	PLGA/collagen I/chitosan	×	√	√	×	[18]
Goats underwent laminectomy	O-PLGA/DF/MAP	×	√	×	×	[19]
Rats with spinal cord injury	Poly(3-hydroxybutyrate-*co*-3-hydroxyvalerate)/poly(lactic acid)/collagen (PHBV/PLA/Col)	×	×	√	×	[20]
Rabbits underwent laminectomy	ECM-inspired micro/nanofibers	×	√	×	×	[21]

PLCL, poly(l-lactide-*co*-ε-caprolactone); RAPA, rapamycin; Th17/Treg cell, T helper 17/regulatory T cell; PLA, poly(l-lactide); ECM, endothelial cell medium; PLGA, poly(lactide-*co*-glycolide); O-PLGA, oriented poly(lactide-co-glycolide); DF, dermal fibroblast; MAP, mussel adhesive protein.

The concentration-dependent safety profile of PLCL-RAPA membranes reveals critical therapeutic window. While 8% RAPA exceeded cytotoxicity thresholds (viability < 70%), lower concentrations (1% to 4%) demonstrated excellent biocompatibility in both neurons and glial cells. This safety profile was corroborated by in vivo survival analysis, in which the 4% RAPA showed optimal outcomes with 100% survival versus 75% in the 8% RAPA groups. Furthermore, the 4% RAPA group achieved remarkable neurorestoration, reducing neuronal loss and increasing neurofilament expression versus TBI controls. The PLCL scaffold alone produced modest improvements in some parameters, which may reflect its physical barrier function, local structural support, or biocompatible modulation of the injury microenvironment. However, the consistently greater therapeutic effects observed in the PLCL-4% RAPA group compared with the PLCL-only group support an additional pharmacological contribution of RAPA release. It should be noted that the 4% concentration was empirically determined on the basis of functional outcomes and biocompatibility rather than a full pharmacokinetic (PK) profile; the absence of such PK data is a limitation of the present study. In addition, the initial sample size was 8 animals per group, and the 8% RAPA group experienced a 25% mortality rate, resulting in a lower effective sample size for behavioral tests such as the Y-maze. This limits the robustness of the conclusions drawn from this cohort and highlights the need for future studies with larger sample sizes reduce mortality. Notably, systemic assessments revealed that RAPA loading mitigated PLCL-induced inflammatory responses (normalized procalcitonin levels; Table S1) and coagulation abnormalities (restored activated partial thromboplastin time values; Table S2), suggesting RAPA’s pleiotropic effects in counteracting polymer-induced foreign body reactions. While no delayed inflammation or coagulation abnormalities were observed up to 21 d, the long-term fate (60 to 90 d) of the PLCL scaffold after RAPA depletion remains to be investigated in future studies. Furthermore, the liver and kidney function tests in TBI rats treated with PLCL-4% RAPA nanofibers demonstrated no statistically significant evidence of hepatotoxicity or nephrotoxicity (Table S3). Importantly, while the PLCL group showed a partial normalization of UREA levels, the RAPA group did not exhibit a significant reduction compared with the TBI group. We have therefore restricted our interpretation of these data to systemic safety assessment, rather than therapeutic efficacy (Table S3).

The scaffold’s ability to prevent postoperative adhesion stems from RAPA’s dual action on fibroblasts dynamics. At the optimal 4% concentration, RAPA significantly reduced Ki67^+^/collagen I^+^ cells, effectively controlling endothelial cell medium (ECM) deposition while maintaining physiological collagen levels. This selective inhibition avoids the risks of underhealing associated with excessive suppression and addresses the key clinical limitation of current commercial dura substitutes that often provoke pathological scarring.

Beyond immunomodulation, this study is among the first to demonstrate the role of a bioactive dura scaffold in preserving meningeal lymphatic vessel, which is a structure increasingly recognized as pivotal for central nervous system waste clearance and neuroimmune communication [22,23]. VEGFR3, encoded by the FLT4 (fms-related receptor tyrosine kinase 4) gene, was the first lymphatic-specific growth factor receptor to be identified and plays a pivotal role in lymphangiogenesis [24,25]. During early embryonic development, VEGFR3 is expressed in developing venous and lymphatic endothelial cells, whereas in normal adult tissues, its expression becomes predominantly restricted to lymphatic endothelial cells [26,27]. Substantial evidence has demonstrated that the Vegfr3 gene is indispensable for the early development of lymphatic vessels, as genetic ablation results in severe lymphatic malformations [28–30]. Furthermore, VEGFR3 has been shown to be crucial for maintaining lymphatic homeostasis postmaturation. Notably, meningeal lymphatic vessels require sustained VEGFR3 signaling for their maintenance, and receptor depletion may lead to lymphatic regression [31]. LYVE-1, a type I integral membrane glycoprotein, serves as both a cell surface marker for lymphatic endothelial cells and a key mediator in hyaluronan metabolism and macromolecular transport [32–34]. While prior investigations have established RAPA’s inhibitory effects on lymphatic endothelial cell proliferation [35,36], the context of TBI involves a disrupted, inflamed dural lymphatic network that may require controlled remodeling rather than robust new growth. It is plausible that excessive, dysfunctional proliferation exacerbates leakage and fibrosis, whereas moderate mTOR inhibition by 4% RAPA tempers this response without abolishing necessary repair. Our results showed no significant inhibition at low doses, suggesting that the observed in vivo lymphatic preservation is not due to direct RAPA toxicity on lymphatics.

Although RAPA treatment significantly reduced aberrant lymphangiogenesis (evidenced by decreased Ki67^+^/VEGFR3^+^ cell populations), it preserved vessels integrity and marker expression (LYVE-1 and VEGFR3), as well as in vitro tube-forming capacity. This indicates that PLCL-RAPA scaffolds selectively inhibited pathological proliferation while preserving the essential lymphatic structure. This dual action (limiting excessive fibroblasts and lymphatic proliferation while preserving meningeal lymphatic integrity) suggests a novel approach to managing scarring and neuroinflammation after TBI. Notably, the in vitro tube formation assay is a simplified model that does not capture flow or immune-lymphatic cross-talk; that experiment was intended only to test whether RAPA directly impairs HLEC tube-forming capacity at the concentrations used. More physiologically relevant models (e.g., coculture with immune cells or flow conditions) are needed to fully understand the mechanisms. Moreover, a time-course analysis (days 3, 7, 14, and 21) would clarify whether RAPA delays or merely regulates lymphatic recovery. In addition, PLCL-RAPA-regulated lymphocyte-derived signals supported lymphatic endothelial maturation and tube-forming capacity in vitro, whereas IL-17A-associated inflammatory signaling partially counteracted these effects. However, future studies using in vivo immune cell depletion or cytokine neutralization strategies will be required to determine the precise contribution of lymphocyte-derived signals to meningeal lymphatic remodeling after TBI. The scaffold’s lymphatic-preserving role likely synergizes with its immune-balancing effects to create a permissive environment for neuronal repair. However, definitive establishment of directional relationships (e.g., whether immune changes drive lymphatic preservation or vice versa) requires future mechanistic studies that provide direct causal evidence for the proposed “meningeal–lymphatic–immune triad”.

In addition, preserving meningeal lymphatic integrity may have profound implications for chronic traumatic encephalopathy. The glymphatic–lymphatic axis couples parenchymal waste clearance, thereby enabling removal of neurotoxic metabolites such as p-tau [37]. TBI impairs aquaporin-4-dependent glymphatic exchange, while persistent neuroinflammation and cervical lymphatic stasis further compromise meningeal drainage, trapping p-tau aggregates [38,39]. By normalizing the morphology of meningeal lymphatic network, our PLCL-RAPA scaffold may therefore directly alleviate parenchymal p-tau accumulation, break the cycle of inflammation-driven waste retention, and offer a protective strategy against the transition from acute TBI to chronic traumatic encephalopathy.

TBI triggers a multifaceted cascade of metabolic, inflammatory, and structural disturbances that compromise both brain parenchyma and meningeal homeostasis [40–42]. By integrating targeted metabolomic profiling of brain tissue and CSF, our study uncovers a coupled metabolic collapse involving mitochondrial dysfunction and inflammatory tryptophan catabolism—a dual signature that underscores the vulnerability of the brain–CSF interface following trauma. One of the most striking findings is the concerted suppression of the TCA cycle in brain tissue after TBI, evidenced by diminished levels of key intermediates including citric acid, isocitric acid, and succinic acid. These changes reflect a profound impairment of oxidative phosphorylation, consistent with known effects of mechanical injury on mitochondrial membrane potential and ATP synthesis. This energetic crisis may compromise neuronal viability and predispose glial cells to reactive activation and neuroinflammation. Moreover, the observed metabolic shifts show that RAPA treatment may reduce the levels of kynurenine, nicotinic acid, and nicotinamide, suggesting a potential role in suppressing neuroinflammation and restoring immune-metabolic balance.

It has been reported that kynurenine, by interacting with the aryl hydrocarbon receptor in CD4^+^ T cells, is associated with the expansion of Treg cells [43]. In contrast, nicotinamide enhances Treg cell differentiation by promoting the acetylation of Foxp3 [44]. A central novelty of this study lies in the scaffold’s ability to reshape the adaptive immune milieu. RAPA loading rebalanced T cell polarization, promoting Treg cell responses while suppressing pathogenic Th17 cell subsets. Future studies using primary CD4^+^ T cells with appropriate polarization protocols are required to directly validate the effects of RAPA on Th17/Treg cell differentiation.

By integrating adaptive immunoregulation with normalization of the meningeal lymphatic network, the PLCL-RAPA scaffold addresses 2 underappreciated but mechanistically intertwined drivers of secondary injury progression. Our scaffold interrupts this feedback loop through localized, controlled RAPA release, positioning it as a versatile platform for advanced dura repair. Further research should explore (a) long-term lymphatic network maturation, (b) cross-talk between meningeal lymphatics and neurogenesis, and (c) synergistic effects with rehabilitation therapies. The scaffold’s modular design allows for incorporation of additional bioactive factors to address these research directions.

This multifunctional scaffold represents a substantial advancement over current dural repair strategies, offering integrated solutions for normalizing the morphology of the meningeal lymphatic network, modulating immunity, and protecting neural tissue—critical requirements for improving TBI outcomes. Our findings position PLCL-RAPA membranes as a promising candidate for clinical translation in neurosurgery and neurotrauma management.

This study successfully demonstrates that electrospun PLCL-RAPA composite membranes represent a substantial advancement in dural repair and neuroprotection for TBI. By incorporating RAPA into a PLCL nanofibrous scaffold, we achieved a multifunctional biomaterial that combines structural support with controlled drug release, offering superior biocompatibility, immunomodulation, and neurorestorative effects. The optimized 4% RAPA formulation exhibited dose-dependent efficacy in restoring immune homeostasis, promoting Treg cell responses, suppressing proinflammatory Th17 cell polarization, which, in turn, mitigated secondary neuronal injury and improved functional recovery. In addition, the material effectively reduced fibrosis and lymphatic endothelial hyperplasia without compromising lymphatic vessel integrity, addressing key challenges in postsurgical adhesion prevention. Importantly, PLCL-RAPA membranes not only enhanced dural regeneration but also attenuated systemic inflammatory responses, as evidenced by normalized coagulation and inflammatory markers. These findings underscore the therapeutic potential of RAPA-loaded scaffolds as an integrated solution for TBI management, combining mechanical repair with pharmacological neuroprotection. Future investigations will focus on elucidating mTOR-dependent signaling pathways in greater detail and optimizing clinical translation strategies, including long-term safety assessments and combinatorial therapeutic approaches. This work establishes a foundation for next-generation bioactive dural substitutes that go beyond passive sealing to actively promote neural recovery.

## Methods

### Preparation of dural patch by electrospinning

The PLCL [poly(l-lactide):poly(ε-caprolactone) = 70:30; average molecular weight = 47,000] was dissolved in hexafluoropropanol (Energy Chemical Company) with a concentration of 10%. Then, this solution was mixed with RAPA (North China Pharmaceutical Company) by mass ratio to prepare spinning solutions with RAPA concentrations of 0%, 1%, 2%, 4%, and 8%. The spinning solution was loaded into a 10-ml syringe and extruded through a 22-gauge needle under 18 kV at a feeding rate of 0.35 mm/min. The fabrication environment was maintained at 20 °C with a relative humidity of 40%. The resulting patch was vacuum-dried at room temperature to remove residual solvents.

### Microstructure characterization

#### Water contact angle

The surface hydrophilicity of the samples was evaluated using the DataPhysics OCA Contact Angle System by dropping 1 μl of deionized water onto the patch surface through a microsyringe. Three samples for each group were measured and analyzed using SCA20 software.

#### Swelling ratio

The swelling properties of the samples were determined according to a previous study [45]. The samples were immersed in phosphate-buffered saline (PBS; pH 7.4, 25 °C) for different time points and then were weighed. Three samples for each group were measured. The percentage of swelling was calculated according to the following equation:

$$\text{Swelling ratio}\;\left(\%\right)=\left[\left(W_{t}-W_{0}\right)\right]/W_{0}\times 100\%$$ (1)

where *W*_0_ is the initial dry mass and *W*_*t*_ as the mass after swelling at time *t*.

### FTIR spectroscopy analysis

The FTIR spectroscopy (OMNI-Sampler Smart Accessory, Nicolet, Bruker, Germany) was used to investigate the chemical bonding of the samples, and the spectra were collected by averaging 64 scans at a resolution of 4 cm^−1^ from 400 to 4,000 cm^−1^.

### XPS analysis

XPS was carried out to determine the quantitative and qualitative elemental surface composition (Ultra DLD spectrometer, Kratos Analytical Ltd., UK) with a monochromatized Al Ka x-ray source (*hν* = 1,486.6 eV). The anode voltage and current were 15 kV and 10 mA, respectively.

### Scanning electron microscope

The surface morphology of the membranes was investigated by SEM (JEOL 5910 LV, Oxford Instruments) at an acceleration voltage of 15 kV. All the samples were gold-coated before SEM analysis.

### Cell culture and drug treatment

Human neuroblastoma SH-SY5Y cells (American Type Culture Collection, Manassas, VA, USA), N9 microglia cells (American Type Culture Collection, Manassas, VA, USA), C8-D1A astrocytes (American Type Culture Collection, Manassas, VA, USA), and NIH/3T3 fibroblasts cells (EallBio, Beijing, China) were maintained in DMEM (Gibco, Gaithersburg, MD, USA) supplemented with 10% fetal bovine serum (FBS; Gibco, Gaithersburg, MD, USA) and 1% penicillin/streptomycin (PS) solution (Gibco, Gaithersburg, MD, USA). HLECs (EallBio, Beijing, China) were maintained in the ECM (ScienCell, CA, USA) + 5% FBS (Gibco, Gaithersburg, MD, USA) + 1% PS (Gibco, Gaithersburg, MD, USA) + 1% endothelial cell growth supplement (ScienCell, CA, USA). Cells were incubated at 37 °C in 5% CO_2_ in humidified air, with the culture medium changed every 2 d. Assays were performed when the culture reached 75 to 80% confluence. The various groups of materials were immersed in DMEM or RPMI 1640 medium for 72 h to prepare the extract solution. Then, the extract solution was diluted to create different concentrations of 10%, 50%, and 90%. The cell samples were incubated for 24 h with extracts of different concentrations.

### Oxygen and glucose deprivation/reoxygenation

Cells were subjected to combined glucose and oxygen deprivation by culturing in glucose-free DMEM medium for 3 h within an anaerobic chamber (Labtron Equipment Ltd., Camberley, Surrey, UK; atmosphere: 95% N_2_, 5% CO_2_). Then, the cell culture medium was replaced with DMEM supplemented with PLCL-RAPA (0 to 8 wt%), 10% FBS, and 1% PS, and the cells were cultured for an additional 24 h at 37 °C in a 5% CO_2_ atmosphere to simulate reperfusion conditions.

### CCK-8 assay

The Cell Counting Kit-8 (CCK-8) assay was performed using a CCK-8 kit (Beyotime, Shanghai, China) following the manufacturer’s protocol. After drug treatment, 10^4^ of cells were placed into a 96-well plate (100 μl per well), and 10 μl of CCK-8 solution was added into each well. After incubating for 3 h, the optical density (OD) value was determined by the Microplate Reader (Thermo Fisher Scientific, Waltham, MA, USA) at 450 nm. The formula for calculating the cell viability was as follows: (mean OD treated wells) / (mean OD control wells) × 100%.

### TUNEL assay

The TUNEL assay was performed using the TMR TUNEL cell apoptosis detection kit (Servicebio, Wuhan, China) following the manufacturer’s protocol. After drug treatment, cells were fixed in 4% formaldehyde, permeabilized with 0.3% Triton X-100 (Solarbio, Beijing, China) for 30 min and then blocked with 3% normal donkey serum (Servicebio, Wuhan, China) for 1 h. After washing with PBS, the cells were then incubated with the TUNEL reaction mixture for 1 h at 37 °C.

### Tube formation assay

The effect of different electrospun micro/nanofibers on angiogenesis of HLECs was assessed by tube formation assay as described before [46]. The 24-well plates were coated with Matrigel (200 μl per well; Corning Matrigel, NY, USA) at 37 °C and 5% CO_2_ for 30 min. Upon gel solidification, HLECs (1 × 10^4^ cells/cm^2^) were seeded onto precoated Matrigel. Then cells were incubated at 37 °C and 5% CO_2_, and images were taken after 12 h. The capillary-like structures of HLECs were photographed with a microscope and analyzed via ImageJ software (National Institutes of Health).

### Animal study

The experimental protocol has been approved by the Animal Welfare Committee of Capital Medical University. Healthy specific-pathogen-free (SPF)-grade Sprague–Dawley rats were purchased from SPF (Beijing) Biotechnology Co. Ltd., with a body weight of 220 to 250 g. They were housed in the SPF-grade animal facility at Capital Medical University. Animal procedures strictly complied with the guidelines outlined in the Guide for the Care and Use of Laboratory Animals, published by the National Institutes of Health. The experimental animals were housed in an environment with a 12-h light/12-h dark cycle, at a temperature of 20 to 24 °C, with ample food and water provided for ad libitum feeding. Fifty-six male Sprague–Dawley rats were randomly divided into the following 7 groups: (a) sham (skin incision only, no craniectomy, and no TBI), (b) TBI (craniectomy + TBI without any implant), (c) TBI + PLCL (craniectomy + TBI with PLCL scaffold containing no RAPA), (d) TBI + PLCL + 1% RAPA, (e) TBI + PLCL + 2% RAPA, (f) TBI + PLCL + 4% RAPA, and (g) TBI + PLCL + 8% RAPA.

### TBI model

The controlled cortical impact model was used to produce TBI, as described previously [47]. Sprague–Dawley rats were anesthetized with 4.0% to 5.0% enflurane and maintained with 1.5% to 3.5% enflurane in a mixture of 70% N_2_O and 30% O_2_. After anesthesia, the rats were secured in a prone position on a stereotactic apparatus. Fur around the surgical site was shaved using an electric razor to expose the skin, and the skin of the head was disinfected with povidone–iodine and 70% ethanol before surgery. A midline incision was made to expose the sagittal and coronal sutures. The dura mater was exposed by drilling a 5-mm-diameter hole 2.5 mm behind and 2.5 mm to the right of the bregma (meticulous care was taken to protect the integrity of the dura mater). The impact pin was positioned at the origin of the circular bone window, and a 40-g weight was dropped from a height of 20 cm onto a 3-mm-diameter impact pin, causing injury to the right brain tissue. Subsequently, the PLCL material, moistened with physiological saline, was placed on the injured brain tissue, and then the scalp was sutured. The rat was gently positioned on a warming blanket to await the return of consciousness. Upon awakening, it was carefully returned to its cage. In the sham surgery group, after anesthesia and surgical preparation, the skull was carefully opened without inflicting any damage to the brain tissue.

### Foot fault test

The foot fault test was conducted at 1, 3, 5, and 7 d after TBI, as described in previous literature [48]. Rats were placed on a grid with a length of 1 m and a width of 0.4 m, with grid holes measuring 4 cm^2^. Each trial allowed rats to walk freely on the grid runway, and their activities were recorded by a camera. Subsequently, the video was reviewed to document the total number of steps taken by the rat, as well as the instances where either the left forelimb or hindlimb slipped off the grid (faulty steps) throughout the entire walking process. The rate of left limb dysfunction was calculated using the formula: left limb dysfunction rate = (faulty steps / total steps) × 100%.

### Y-maze test

To investigate the effect of different concentrations of RAPA on learning and memory in rats after TBI, the Y-maze experiment was performed as described in previous literature [49]. The Y-maze apparatus, resembling the letter Y, consists of 3 equal-length arms measuring 45 cm in length, 15 cm in width, and 40 cm in height, each separated by 120° angles. The 3 arms were randomly designated as start arm, old arm, and new arm. During the initial trial for training purposes, access to the new arm was restricted, permitting the rat to freely explore the start arm and old arm for 10 min. Subsequently, in the second trial designated for retention testing, the barriers to all 3 arms were removed, allowing the rat unrestricted movement starting from the same start arm for 5 min. The rat was deemed to have entered an arm once all 4 of its limbs have been positioned on the arm’s surface. The rat was recorded and analyzed using the ANY-maze video tracking system (Stoelting Co.). After each trial, the equipment was meticulously sanitized using 75% ethanol to eliminate any residual olfactory cues from the prior test. The percentage of new arm visits (number of new arm entries / total arm entries × 100) and the percentage of time spent in the new arm (total time spent in the new arm / total time spent in all 3 arms × 100) were calculated to reflect the rat’s spatial recognition memory.

### Lymphocyte isolation and flow cytometry analysis

Lymphocytes were isolated according to the manufacturer’s protocol. After isolation, PBS was added to prepare a single-cell suspension. Prior to staining, the single-cell suspension was treated with an Fc blocker (BD Pharmingen, San Diego, CA, USA) to block the Fc receptors. Following the blocking step, the cells were stained with antibodies against CD3 (1:200; BioLegend, San Diego, CA, USA), CD4 (1:200; BioLegend, San Diego, CA, USA), IL-17A (eBioscience, San Diego, CA, USA), and FOXP3 (eBioscience, San Diego, CA, USA). The stained cells were then analyzed using flow cytometry on a BD FACSDiva (BD Biosciences) instrument.

### Targeted metabolomics of tryptophan pathway in CSF

CSF was collected via cisterna magna puncture prior to sacrifice, centrifuged at 3,000*g* for 10 min to remove debris, and stored at −80 °C for subsequent analysis. CSF samples were analyzed using a customized tryptophan metabolism panel, which covers 26 metabolites involved in the kynurenine, serotonin, and nicotinamide pathways. Sample preparation included protein precipitation with cold methanol, followed by liquid chromatography–tandem mass spectrometry (LC-MS/MS) quantification using stable isotope-labeled internal standards. Key metabolites such as quinolinic acid, kynurenine, nicotinic acid, nicotinamide, and 5-hydroxyindoleacetic acid were reliably quantified across all groups.

### Targeted metabolomics of brain energy metabolism

At 21 d after TBI surgery, brain tissue from the perilesional cortex was rapidly dissected on ice, snap-frozen in liquid nitrogen, and stored at −80 °C. Frozen brain tissues were homogenized in 80% methanol, centrifuged, and subjected to targeted LC-MS/MS profiling using a prevalidated energy metabolism panel (BioNovoGene, Suzhou, China). The panel included 15 metabolites related to the glycolytic pathway and TCA cycle, including citric acid, fumaric acid, pyruvic acid, and succinic acid. Chromatographic separation was achieved on a C18 column with multiple reaction monitoring in negative ion mode. Metabolite intensities were normalized to protein content and expressed as nanograms per milligram.

### Western blot

Protein expression analysis was performed following a previously published protocol [50]. Rat brain tissue samples were homogenized in radioimmunoprecipitation assay lysis buffer supplemented with a cocktail of protease and phosphatase inhibitors. After protein quantification, 20 μg of total protein from each sample was separated via 10% SDS-polyacrylamide gel electrophoresis and subsequently electrotransferred to polyvinylidene difluoride membranes.

Membranes were blocked for 30 min at room temperature using a protein-free blocking solution (Servicebio, Wuhan, China) and then incubated overnight at 4 °C with the following primary antibodies: anti-OGDH (1:1,000; Proteintech, Wuhan, China), anti-KMO (1:500; Proteintech, Wuhan, China), anti-p-mTOR (1:500; Proteintech, Wuhan, China), and anti-β-actin (1:1,000; Santa Cruz Biotechnology, CA, USA).

Following primary antibody incubation, membranes were washed and incubated with horseradish-peroxidase-conjugated secondary antibodies (1:1,000; Santa Cruz Biotechnology, CA, USA) for 1 h at room temperature. Protein bands were visualized using an enhanced chemiluminescence detection kit (Millipore, MA, USA). Band intensities were quantified using ImageJ software (National Institutes of Health, MD, USA), and relative protein levels were calculated after normalization to β-actin as the internal control.

### Histological assessment

At 21 d after TBI surgery, rats were anesthetized with an intraperitoneal injection of sodium pentobarbital (50 mg/kg) and perfused through the left cardiac ventricle with 0.9% saline. They were decapitated, and their brains were preserved in a 10% solution of neutral-buffered formaldehyde (Servicebio, Wuhan, China). Subsequently, the preserved brains were sectioned in a coronal plane and readied for the process of paraffin impregnation. The paraffin blocks were sectioned (7 μm) for HE staining. Then, HE staining was performed using an HE staining kit (Servicebio, Wuhan, China) according to the manufacturer’s instructions.

### Immunofluorescence staining

Immunofluorescence staining was conducted as previously described [51]. After 24 h of drug treatment, the cells were washed with PBS (Servicebio, Wuhan, China) and then fixed with 4% paraformaldehyde for 30 min. Subsequently, the cells were permeabilized with 0.3% Triton X-100 (Solarbio, Beijing, China) for 30 min and then blocked with 3% normal donkey serum (Servicebio, Wuhan, China) for 1 h at room temperature. Next, cells were incubated with the following specific primary antibodies in a moist chamber for 12 h at a temperature of 4 °C: anti-neuronal nuclei (NeuN) (1:200; Millipore, CA, USA), anti-LYVE-1 (1:200; Proteintech, Wuhan, China), anti- VEGFR3 (1:200; Abcam, Cambridge, MA, USA), anti-collagen I (1:200; Proteintech, Wuhan, China), anti-Ki67 (1:200; BioLegend, CA, USA), anti-CD3 (1:200; BioLegend, San Diego, CA, USA), anti-CD4 (1:200; BioLegend, San Diego, CA, USA), anti-IL-17A (eBioscience, San Diego, CA, USA), and anti-FOXP3 (eBioscience, San Diego, CA, USA). Afterward, they were treated with secondary antibodies (1:200; Santa Cruz Biotechnology, CA, USA) that were conjugated to fluorescent markers.

For immunofluorescence staining of tissues, paraffin sections were first deparaffinized. Subsequently, the tissue sections were immersed in fixation trays filled with citrate buffer for antigen retrieval at a pH of 6.0. These were then heated in a microwave at a temperature of 95 °C for a duration of 20 min to enhance the retrieval of antigens.

The sections were subsequently washed 3 times with PBS and blocked with 3% donkey serum for 60 min. Then, the sections were incubated with primary antibodies overnight at 4 °C. The following primary antibodies were used: anti-LYVE-1 (1:200; Proteintech, Wuhan, China), anti-VEGFR3 (1:200; Abcam, Cambridge, MA, USA), anti-collagen I (1:200; Proteintech, Wuhan, China), anti-neurofilament 200 (NF200; 1:200, Abcam, Cambridge, MA, USA), anti-CD4 (1:200, BioLegend, San Diego, CA, USA), anti-IL-17A (eBioscience, San Diego, CA, USA), and anti-FOXP3 (eBioscience, San Diego, CA, USA). Subsequently, they were incubated with secondary antibodies (1:200; Santa Cruz Biotechnology, CA, USA) conjugated with fluorescent markers.

### Rescue experiment

Mouse venous blood was collected into anticoagulant-treated tubes. One milliliter of whole blood was carefully layered onto 1.6 ml of mouse lymphocyte separation medium and centrifuged at 450*g* for 30 min at room temperature. After centrifugation, the mononuclear lymphocyte layer at the interface was carefully collected, washed, and resuspended in complete lymphocyte culture medium. The isolated lymphocytes were stimulated with phytohemagglutinin and subsequently treated with DMEM, PLCL extract, or PLCL-4% RAPA extract under the same conditions described above. After incubation, the culture supernatants were collected and centrifuged to remove cellular debris. The resulting lymphocyte-conditioned media were used for subsequent SVEC4-10 endothelial cell experiments.

For the IL-17A rescue experiment, recombinant IL-17A was added to conditioned medium from the PLCL-4% RAPA group at a final concentration of 50 ng/ml before treatment of SVEC4-10 cells. SVEC4-10 cells were used as a murine lymph-node-derived endothelial cell model. Cells were maintained in complete endothelial growth medium and switched to low-growth-factor endothelial medium before conditioned medium treatment. Endothelial-maturation-related markers were evaluated by immunofluorescence staining for LYVE-1 and VEGFR3, and proliferative status was assessed by Ki67 staining. For tube formation assays, SVEC4-10 cells were seeded onto Matrigel-coated plates and cultured with the corresponding conditioned media. Tube-like networks were imaged under a phase-contrast microscope and quantified for tube area fraction, total tube length, junction number, and mesh number.

### Statistical analysis

Raw LC-MS/MS data were processed with vendor-specific software and exported into CSV format. Data normalization and quality control were performed using MetaboAnalyst 5.0. Group means were calculated for each metabolite, and differential abundance was assessed using log_2_FC relative to the TBI group. For selected metabolites, group comparisons were visualized via boxplots and heatmaps using Seaborn and Matplotlib in Python 3.10. Pathway enrichment analysis was performed based on Kyoto Encyclopedia of Genes and Genomes (KEGG) annotations using manual curation. Metabolites involved in the TCA cycle, glycolysis, and tryptophan catabolism were further analyzed for mechanistic interpretation. All results were expressed as means ± SD. GraphPad Prism 7.0 and SPSS 17.0 for Windows were used for statistical analysis. For comparisons between 2 groups, Student’s *t* tests or Mann–Whitney *U* tests were used. For multiple groups, one-way analysis of variance (ANOVA), followed by Tukey’s post hoc test or the Mann–Whitney *U* test with Bonferroni correction was used.

## Data Availability

All relevant data that support the findings of this study are available within the article and Supplementary Materials. All data are available from the corresponding authors upon request. Source data are provided with this paper.
