# Supplementary material for: Lymphatic-Immune Microenvironment Engineering via Rapamycin-Eluting Nanofibers for Meningeal Repair and Neuroprotection in Traumatic Brain Injury Rats
Source: Research (Wash D C). 2026 Sep 14;9:1415. doi: 10.34133/research.1415 (PMC13572802; doi:10.34133/research.1415)
Supplement: Supplementary file 1 [file research.1415.f1.docx]

**Supplementary Materials**

**Fig.S1 The drug release behavior and hemolysis characterization of PLCL-RAPA nanofibrous membranes.** (A) In vitro rapamycin release profile of rapamycin-loaded PLCL nanofibrous membranes. (B) Hemolytic analysis of rapamycin-loaded PLCL nanofibrous membranes. Data are presented as the mean ± SD and analyzed using ANOVA with Tukey post hoc test (***p < 0.001 vs. PLCL group. ###p < 0.001 vs. 1% RAPA group. %%%p < 0.001 vs. 2% RAPA group. $$$p < 0.001 vs. 4%RAPA group).

**Fig.S2 Cytotoxicity assessment of PLCL-RAPA nanofibers.**  Cytotoxicity assay of electrospun PLCL-RAPA nanofibrous membranes with varying RAPA content (0-8 wt%) on neurons (A), astrocytes (B), and microglial cells (C). Data are presented as the mean ± SD and analyzed using ANOVA with Tukey post hoc test (*p < 0.05, **p < 0.01 and p < 0.001 vs. DMEM group. #p < 0.05, ##p < 0.01, and ###p < 0.001 vs. PLCL group. %p < 0.05, %%p < 0.01, and %%%p < 0.001 vs. 1%RAPA group. $p < 0.05, $$p < 0.01 and $$$p < 0.001 vs. 2%RAPA group. &p < 0.05 and &&p < 0.01 vs. 4%RAPA group).

**Figure. S3 Lymphocyte conditioned medium from the RAPA-PLCL enhances lymphatic endothelial maturation and tube formation, while IL-17A attenuates these effects.** (A) Schematic illustration of the in vitro experimental design. (B) Representative immunofluorescence images of primary lymphocytes showing the expression of FOXP3 and IL-17A in the DMEM, PLCL, 4% RAPA, and 4% RAPA + IL-17A groups. (C) Representative phase-contrast images of lymphatic endothelial tube formation on Matrigel after treatment with conditioned medium derived from different lymphocyte groups. (D) Representative immunofluorescence images of lymphatic endothelial cells cultured on Matrigel and stained for LYVE1, Ki67, VEGFR3, and DAPI. (E) Quantification of lymphocyte subsets, including FOXP3⁺/CD4⁺ and IL-17A⁺/CD4⁺ cells. (F) Quantitative analysis of lymphatic endothelial tube formation, including tube area fraction, total tube length, number of junctions, and number of meshes. (G) Quantification of Ki67 expression and lymphatic endothelial marker-positive areas, including LYVE1 and VEGFR3. Data are presented as mean ± SD and analyzed using ANOVA with Tukey post hoc test ( *P < 0.05, **P < 0.01 and ***P < 0.001 vs. DMEM group. #P < 0.05, ##P < 0.01 and ###P < 0.001 vs. PLCL group. &P < 0.05, &&P < 0.01 and &&&P < 0.001 vs. 4% RAPA group).

**Fig.S4 Schematic diagram of experimental model and treatment.**

**Fig.S5 PLCL-RAPA inhibits proliferation of fibroblasts in traumatic brain injury rats.** Representative immunofluorescence images of dura mater distant from the implantation area. Collagen I (green) and DAPI (blue). Scale bars: 20 µm.

**Fig. S6 Expression of KMO, p-mTOR and OGDH in brain tissues of rats after traumatic brain injury.** (A) Expression of KMO detected by western blot; (B) Quantification analysis of KMO analyzed by western blot. (C) Expression of p-mTOR detected by western blot; (D) Quantification analysis of p-mTOR analyzed by western blot. (E) Expression of OGDH detected by western blot; (F) Quantification analysis of OGDH analyzed by western blot.

**Table S1. Routine blood test for TBI rats**

|  | **Sham (n=5)** | **TBI (n=5)** | **PLCL (n=5)** | **4% RAPA (n=5)** | **p** |
| --- | --- | --- | --- | --- | --- |
| **WBC (10^9/L) (median [IQR])** | 6.91 [6.89, 7.09] | 6.28 [6.02, 7.04] | 5.95 [4.77, 6.15] | 7.13 [6.92, 7.38] | 0.144 |
| **Neu% (mean (SD))** | 10.48 (5.66) | 13.90 (7.19) | 13.66 (3.93) | 16.32 (5.81) | 0.480 |
| **Lym% (mean (SD))** | 80.52 (7.97) | 78.78 (6.38) | 78.84 (8.66) | 75.80 (6.02) | 0.784 |
| **Mon% (mean (SD))** | 8.74 (3.61) | 6.90 (2.74) | 7.10 (5.52) | 7.44 (2.31) | 0.865 |
| **Eos% (median [IQR])** | 0.20 [0.00, 0.20] | 0.30 [0.10, 0.30] | 0.30 [0.10, 0.30] | 0.30 [0.20, 0.50] | 0.485 |
| **Bas% (median [IQR])** | 0.10 [0.10, 0.10] | 0.20 [0.10, 0.20] | 0.10 [0.10, 0.10] | 0.10 [0.10, 0.10] | 0.160 |
| **NeuT (10^9/L) (median [IQR])** | 0.53 [0.37, 0.77] | 0.85 [0.62, 0.91] | 0.71 [0.59, 0.74] | 0.97 [0.93, 1.18] | 0.212 |
| **LymT (10^9/L) (mean (SD))** | 5.69 (1.65) | 5.10 (1.04) | 4.39 (1.31) | 5.36 (0.38) | 0.388 |
| **MonT (10^9/L) (mean (SD))** | 0.61 (0.29) | 0.43 (0.15) | 0.36 (0.27) | 0.52 (0.16) | 0.364 |
| **EosT (10^9/L) (median [IQR])** | 0.01 [0.00, 0.02] | 0.02 [0.01, 0.02] | 0.02 [0.01, 0.02] | 0.02 [0.01, 0.04] | 0.518 |
| **BasT (10^9/L) (median [IQR])** | 0.01 [0.01, 0.01] | 0.01 [0.01, 0.01] | 0.01 [0.01, 0.01] | 0.01 [0.01, 0.01] | 0.392 |
| **Aly% (mean (SD))** | 7.34 (1.85) | 7.50 (2.45) | 9.88 (3.15) | 6.80 (2.64) | 0.274 |
| **AlyT (10^9/L) (median [IQR])** | 0.43 [0.41, 0.49] | 0.40 [0.36, 0.63] | 0.52 [0.49, 0.52] | 0.46 [0.36, 0.51] | 0.819 |
| **LIC% (median [IQR])** | 0.00 [0.00, 0.00] | 0.10 [0.10, 0.60] | 0.00 [0.00, 0.20] | 0.40 [0.10, 0.70] | 0.330 |
| **LIC (10^9/L) (median [IQR])** | 0.00 [0.00, 0.00] | 0.01 [0.01, 0.03] | 0.00 [0.00, 0.01] | 0.03 [0.01, 0.05] | 0.263 |
| **NRBC% (median [IQR])** | 0.84 [0.30, 0.91] | 0.22 [0.09, 0.38] | 0.10 [0.00, 0.29] | 0.28 [0.23, 0.32] | 0.361 |
| **NRBC (10^9/L) (median [IQR])** | 0.04 [0.02, 0.06] | 0.01 [0.01, 0.02] | 0.01 [0.00, 0.01] | 0.02 [0.02, 0.02] | 0.313 |
| **RBC (10^12/L) (median [IQR])** | 7.23 [7.17, 7.41] | 6.62 [5.96, 6.97] | 6.79 [6.54, 6.92] | 6.90 [6.90, 7.59] | 0.417 |
| **HGB (g/L) (median [IQR])** | 140.00 [139.00, 147.00] | 126.00 [117.00, 143.00] | 131.00 [130.00, 135.00] | 139.00 [135.00, 146.00] | 0.392 |
| **HCT % (median [IQR])** | 40.00 [39.20, 42.00] | 36.20 [35.10, 40.20] | 38.70 [37.30, 38.90] | 39.80 [38.70, 41.80] | 0.479 |
| **MCV (fL) (mean (SD))** | 55.96 (1.54) | 56.62 (1.87) | 57.24 (1.09) | 56.16 (0.95) | 0.505 |
| **MCH (pg) (mean (SD))** | 19.58 (0.44) | 19.70 (0.56) | 19.34 (0.42) | 19.42 (0.44) | 0.633 |
| **MCHC (g/L) (median [IQR])** | 350.00 [349.00, 350.00] | 348.00 [345.00, 354.00] | 340.00 [337.00, 348.00] | 348.00 [347.00, 349.00] | 0.151 |
| **RDW-CV% (median [IQR])** | 20.40 [20.20, 20.80] | 15.80 [15.30, 16.30] | 17.00 [16.10, 17.50] | 20.50 [20.40, 21.70] | 0.175 |
| **RDW-SD (fL) (mean (SD))** | 43.84 (5.23) | 37.68 (5.33) | 40.64 (7.58) | 47.20 (3.37) | 0.081 |
| **PLT (10^9/L) (median [IQR])** | 727.00 [671.00, 779.00] | 715.00 [672.00, 726.00] | 816.00 [751.00, 865.00] | 630.00 [611.00, 691.00] | 0.133 |
| **MPV (fL) (mean (SD))** | 7.12 (0.51) | 7.04 (0.44) | 7.74 (0.32) | 6.84 (0.55) | 0.040 |
| **PDW (fL) (mean (SD))** | 9.04 (1.05) | 8.88 (1.06) | 9.96 (0.57) | 8.98 (2.15) | 0.564 |
| **PCT % (median [IQR])** | 0.49 [0.49, 0.54] | 0.49 [0.47, 0.49] | 0.63 [0.58, 0.63] | 0.45 [0.42, 0.46] | 0.004 |
| **P-LCR% (mean (SD))** | 16.28 (3.94) | 15.48 (3.24) | 20.62 (2.55) | 14.86 (4.18) | 0.079 |
| **P-LCC (10^9/L) (median [IQR])** | 114.00 [100.00, 127.00] | 102.00 [101.00, 125.00] | 156.00 [148.00, 173.00] | 85.00 [84.00, 92.00] | 0.016 |

**Table S2. Coagulation function of TBI rats**

|  | **Sham (n=5)** | **TBI (n=5)** | **PLCL (n=5)** | **4% RAPA (n=5)** | **p** |
| --- | --- | --- | --- | --- | --- |
| **APTT.s. (mean (SD))** | 26.68 (3.42) | 29.44 (7.90) | 40.88 (3.29) | 31.49 (2.67) | 0.002 |
| **PT.s. (mean (SD))** | 10.26 (0.84) | 11.20 (0.63) | 11.46 (0.42) | 11.56 (0.41) | 0.014 |
| **INR (mean (SD))** | 1.05 (0.10) | 1.02 (0.08) | 1.02 (0.07) | 0.99 (0.03) | 0.690 |
| **FIBC.g.L. (mean (SD))** | 1.54 (0.20) | 1.41 (0.09) | 1.52 (0.15) | 1.66 (0.06) | 0.073 |

**Table S3. Liver and kidney function tests for TBI rats**

|  | **Sham (n=6)** | **TBI (n=6)** | **PLCL (n=6)** | **4% RAPA (n=6)** | **p** |
| --- | --- | --- | --- | --- | --- |
| **ALT (median [IQR])** | 59.89 [53.30, 65.60] | 60.74 [57.98, 93.13] | 57.80 [51.72, 64.81] | 62.25 [56.92, 68.38] | 0.652 |
| **AST (mean (SD))** | 183.89 (38.23) | 273.26 (82.38) | 237.18 (41.87) | 230.76 (26.55) | 0.054 |
| **ALP (mean (SD))** | 260.42 (67.92) | 276.25 (76.79) | 244.10 (81.92) | 339.30 (48.33) | 0.129 |
| **TP (mean (SD))** | 58.10 (5.46) | 51.79 (4.11) | 56.40 (4.58) | 55.73 (3.58) | 0.129 |
| **ALB (mean (SD))** | 31.53 (2.25) | 29.74 (1.72) | 31.91 (2.45) | 31.44 (1.52) | 0.277 |
| **GLB (mean (SD))** | 26.56 (3.50) | 22.06 (2.66) | 24.49 (2.67) | 24.29 (2.21) | 0.081 |
| **A/G (mean (SD))** | 1.20 (0.11) | 1.36 (0.12) | 1.31 (0.11) | 1.30 (0.08) | 0.087 |
| **CREA (median [IQR])** | 44.21 [41.24, 47.26] | 59.68 [51.75, 78.83] | 46.45 [42.69, 50.31] | 44.56 [40.53, 51.92] | 0.100 |
| **UREA (median [IQR])** | 6.79 [6.22, 7.21] | 8.16 [7.82, 8.78] | 7.29 [6.95, 7.63] | 8.18 [7.62, 8.41] | 0.006 |
| **UA (median [IQR])** | 105.44 [84.03, 118.76] | 125.28 [106.09, 135.03] | 117.61 [96.78, 136.44] | 103.53 [102.06, 111.61] | 0.813 |
| **TBIL (mean (SD))** | 381.50 (236.22) | 425.24 (316.14) | 436.93 (249.30) | 438.77 (136.96) | 0.974 |
| **GGT (mean (SD))** | 3.07 (1.88) | 2.74 (0.75) | 2.64 (0.65) | 1.95 (0.52) | 0.368 |
| **TBA (median [IQR])** | 7.64 [6.67, 9.98] | 11.35 [8.62, 13.25] | 9.10 [8.22, 10.42] | 8.10 [6.42, 18.40] | 0.552 |
| **PA (median [IQR])** | 2991.90 [2802.60, 3351.83] | 3005.15 [2846.60, 3165.50] | 3898.15 [3649.80, 4375.18] | 3019.45 [2785.12, 3215.90] | 0.394 |
| **CHE (median [IQR])** | 114.00 [102.35, 124.30] | 101.20 [83.53, 112.80] | 100.10 [94.15, 108.45] | 85.50 [81.05, 110.80] | 0.146 |
| **Serum ammonia (mean (SD))** | 206.54 (28.77) | 229.76(39.45) | 237.05 (63.76) | 214.63 (35.48) | 0.643 |
| **DBIL (median [IQR])** | 57.68 [54.92, 62.39] | 67.17 [60.51, 74.46] | 60.72 [55.54, 65.44] | 46.57 [40.87, 62.57] | 0.397 |
